# The Reciprocal Interaction Between LncRNA CCAT1 and miR-375-3p Contribute to the Downregulation of IRF5 Gene Expression by Solasonine in HepG2 Human Hepatocellular Carcinoma Cells

**DOI:** 10.3389/fonc.2019.01081

**Published:** 2019-10-18

**Authors:** Zheng Liu, ChangJu Ma, XiaoJuan Tang, Qing Tang, LiJie Lou, Yaya Yu, Fang Zheng, JingJing Wu, Xiao-bo Yang, Wei Wang, Swei Sunny Hann

**Affiliations:** ^1^Laboratory of Tumor Biology, The Second Clinical College of Guangzhou University of Chinese Medicine, Guangzhou University of Chinese Medicine, Guangzhou, China; ^2^Department of Gastrointestinal Surgery, The Second Clinical College of Guangzhou University of Chinese Medicine, Guangzhou University of Chinese Medicine, Guangzhou, China; ^3^Guangdong Provincial Key Laboratory of Clinical Research on Traditional Chinese Medicine Syndrome, The Second Clinical College of Guangzhou University of Chinese Medicine, Guangzhou University of Chinese Medicine, Guangzhou, China

**Keywords:** solasonine, HCC, IRF5, lncRNA CCAT1, miR-375-3p, SP1

## Abstract

Solasonine (SS), a natural glycoalkaloid component, has been shown to have potent inhibitory activity and cytotoxicity against many cancer types. However, the precise mechanisms underlying this, particularly in hepatocellular carcinoma (HCC) are poorly understood. In this study, we showed that SS inhibited growth of HCC cells. Mechanistically, we observed that SS increased the expression of miR-375-3p, whereas reducing levels of long non-coding RNAs (lncRNAs) CCAT1 was noticed in HepG2 HCC and other cells. In addition, we found that SS repressed transcription factors, SP1 and interferon regulatory factor 5 (IRF5), protein expressions. There was a reciprocal interaction among miR-375-3p, CCAT1, and SP1. Moreover, SS inhibited IRF5 promoter activity, which was not observed in cells transfected with excessive expressed SP1 vectors. Interestingly, exogenously expressed IRF5 was shown to reverse expressions of SS-inhibited CCAT1 and induced-miR-375-3p; and neutralized SS-inhibited growth of HCC cells. Similar results were also found *in vivo* mouse model. Collectively, our results show that SS inhibits HepG2 HCC growth through the reciprocal regulation between the miR-375-3p and lncRNA CCAT1, and this results in transcription factor SP1-mediated reduction of IRF5 expression. The regulations and interactions among miR-375-3p, CCAT1, SP1, and IRF5 axis unveil a novel molecular mechanism underlying the anti-HCC growth by SS. IRF5 may be a potential target for treatment of HCC.

## Introduction

Hepatocellular carcinoma (HCC) is one of the most common malignancies with high frequencies of recurrence and metastasis. The treatment of HCC requires multidisciplinary treatment modalities (1). Although substantial treatment improvements have been made, HCC still remains to have a poor prognosis (1). Currently, there are limited treatment options for advanced HCC. The novel therapeutic advances with several small molecules kinase inhibitors and immunotherapy, such as programmed death-ligand 1 (PD-L1)/programmed cell death 1 (PD-1) pathway, may likely change the treatment scenario of HCC (2, 3). Thus, searching and exploring novel strategies for the treatment of HCC is of great importance.

Numerous anti-cancer agents have been isolated from natural products, such as plants including their semi-synthetic and synthetic derivates. Among these, solasonine (SS), one major glycoalkaloid extracted from *S. lycocarpum* and found *Solanum* species, has been demonstrated anti-proliferative activity against many cancer types (4–7). SS could inhibit cell proliferation, migration and colony formation of glioma cells through targeting the anti-inflammatory molecules, NF-κB and mitogen-activated protein kinase (MAPK) signaling axis cascade (8). Another report investigated the anti-proliferative activity of SS against several cancer types and demonstrated that SS may be a potential anticancer drug candidate (5). Nevertheless, the precise molecular mechanism underlying the anti-cancer effects of SS still remains to be determined.

Long non-coding RNAs (lncRNAs), which lack a complete open reading frame and play an important role in biological processes, have been illustrated to function as important regulators in several biological functions, such as cell proliferation, differentiation and apoptosis, in cancer (9). Many lncRNAs are dysregulated and involved in tumorigenesis, progression, metastasis, prognosis, or diagnosis and even treatment in HCC (10). Among these, the expression of lncRNA, CCAT1, was markedly increased in the HCC tissues compared to that in the pair-matched non-cancerous tissues. CCAT1 promoted the proliferation and migration of HCC cells by functioning as a molecular sponge for miRNA let-7, and led to the control of endogenous targets, such as high-mobility group protein A2 (HMGA2) and c-Myc, suggesting that CCAT1 played a critical role in the growth and progression of HCC via competitively sponging to let-7 (11). In addition, Kaplan–Meier analysis found that the patients with reduced CCAT1 levels showed better overall survival compared to those with increased CCAT1 expression. Moreover, Cox proportional hazards analyses demonstrated that CCAT1 could be used as an independent prognostic indicator in patients with HCC (12). This finding, together with other reports, indicated that the aberrant expression of CCAT1 promoted proliferation, migration and invasion in HCC both *in vivo* and *in vitro* (13). However, the role of CCAT1 and the detailed molecular mechanism underlying the involvement of HCC development and progression still remain unknown.

MicroRNAs (miRNAs) have been involved in many types of diseases, including human cancer. A large body of evidence has demonstrated that miRNAs regulate multiple biological functions, such as cancer cell differentiation, growth, apoptosis and metastasis (14). MiR-375, which acted as a candidate tumor suppressor miRNA, has been showed to suppress growth and induce apoptosis in several cancer types (15–17). Studying the expression of miR-375 and its target gene SMAD family member 7 (SMAD7) polymorphisms (rs4939827) in colorectal cancer (CRC) patients found that there was a significant association between miR-375 and the susceptibility to CRC, and that miR-375 and rs4939827 SNP in SMAD7 could be considered as a potential biomarker for early diagnosis of CRC (18). MiR-375 was among the most downregulated miRNAs in resistant breast cancer cells. Forced expression of miR-375 could sensitize tamoxifen-resistant cells to tamoxifen and reversed epithelial-to-mesenchymal transition (EMT) in breast cancer cells, suggesting that miR-375 might be used for potential therapeutic approaches for the treatment of tamoxifen-resistant breast cancer (19). The lncRNA-miRNA regulatory networks, such as CCAT1 interacted with miRNAs, have been implicated to regulate tumorigenesis and progression in cancers including HCC (11, 14, 20). CCAT1 functions as a molecular regulator for miRNA by competitively sponging, and leading to regulate endogenous target gene expression and subsequent biological function (11, 21, 22).

Transcription factor interferon regulatory factor 5 (IRF5) has been shown to regulate the expression of genes involved in the inflammatory responses and the stimulation of the immune system (23). Moreover, studies have demonstrated that IRF5 negatively regulated cell growth and oncogene activation, favoring cell differentiation, apoptosis, and sensitivity to oncolytic therapy (24–26). IRF5 proved to be an adverse independent prognostic factor for overall survival (OS) and recurrence free survival (RFS) in clear cell renal cell carcinoma (ccRCC) cells (27). On the contrary, IRF5 also acts as a tumor suppressor in several human cancers (28, 29). Thus, the true role of this transcription factor in tumor biology remains to be undetermined. Given the role of IRF5 in pathogenesis, its clinical and prognostic value in cancer, IRF5 may represent a potential therapeutic target for cancer. The connection and interaction of miRNA and IRF5 have also been studied. MiR-146b was shown to target IRF5, resulting in the regulation of macrophage activation (30). miR-let7a also directly targeted pro-inflammatory gene high-mobility group protein A2 (HMGA2), thereby suppressing anti-citrullinated protein antibodies (ACPAs)-induced IRF5 expression through phosphoinositide 3-kinase (PI3-K) signaling in macrophages (31). However, until now, there has been less information demonstrating the links between IRF5 and lncRNA expression and function.

In the current study, we explored the potential mechanism underlying the anti-HCC cell growth by SS. We observed that SS inhibited HCC cell growth through the reciprocal regulation of miR-375-3p and CCAT1; this resulted in transcription factor SP1-mediated inhibition of IRF5 gene expression.

## Materials and Methods

### Reagents and Cell Culture

Liver cancer cell line, HepG2, was obtained from the Cell Line Bank at the Laboratory Animal Center of Sun Yat-sen University (Guangzhou, China), Cell line, QGY-7703, and human normal hepatocyte (LO2) cells were obtained from Cell Bank of Chinese Academy of Science (Shanghai, China). All cell lines had no HCV infection. Monoclonal antibodies against SP1 and IRF5 were purchased from Cell Signaling Technology Inc. (Beverly, MA, USA) and AB Colonel Technology Inc. (Wuhan, China), separately. 3-(4, 5-Dimethylthiazol-2-yl)-2, 5-diphenyltetra-zolium bromide (MTT) powder was provided by Sigma-Aldrich (St. Louis, MO, USA). 5-Ethynyl-2′-deoxyuridine (EdU) detection kit and miR-375-3p mimics were purchased from Ribo Biological Co., Ltd. (Guangzhou, China). Lipofectamine 3000 reagent was obtained from Life Technologies (Carlsbad, CA, USA). The pcDNA3.1 (control vector) and the SP1 overexpression plasmid were kindly provided by Dr. Thomas E. Eling (NIEHS, Research Triangle Park, NC, USA). The detailed information for the critical reagents used was summarized in Table 1 (Supplementary Material). SS was purchased from Chengdu Must Biotechnology Company (Chengdu, Sichuan, China). Cells were cultured at 37°C in 5% CO2 in RPMI-1640 medium (GIBCO, Life Technologies, Grand Island, NY, USA) supplemented with 10% FBS (HyClone, Invitrogen, Camarillo, CA, USA), 100 U/mL penicillin and 100 μg/mL streptomycin (Invitrogen, Carlsbad, CA, USA). In addition, the medium grown HepG2-Luc was added with Geneticin G-418 Sulfate (Life Technologies, Carlsbad, CA, USA) (200 μg/mL).

**Table 1:** The information of critical reagents used in this study.

		**Dilutions**	**Catalog numbers**	**Resources**	**Final concentration**
Antibody	SP1	1:1,000	#9389	Cell Signaling Technology	
	IRF5	1:750	A1149	ABclonal Technology	
	GAPDH	1:20,000	ab128915	Abcam	
	Anti-rabbit IgG HRP-linked	1:2,000	#7074	Cell Signaling Technology	
Plasmids	pcDNA3.1			Dr. Thomas E. Eling	0.2 μg/mL
	EX-NEG-M02		EX-NEG-MO2	GeneCopoeia	0.2 μg/mL
	pcDNA3.1-SP1			Dr. Thomas E. Eling	2.0 μg/mL
	EX-NEG-M02-IRF5		EX-Z4372-MO2	GeneCopoeia	1.5 μg/mL
	EX-NEG-M02-CCAT1		CS-GS3356-MO2	GeneCopoeia	0.5 μg/mL
	pEZX-PL01-IRF5 promoter		HPRM33965-PL01	GeneCopoeia	1.0 μg/mL
NC/mimics	NC		miR 01101	Ribo Biological Co., Ltd.	100 nM
	miR-375-3p mimics		miR 10005307	Ribo Biological Co., Ltd.	100 nM

### Cell Viability Assay

HepG2 and QGY-7703 cells (3–5 x 10^3^ cells/well) and normal hepatocyte (LO2) cells (3–5 x 10^3^ cells/well) were seeded into a 96-well microtiter plate and treated with increasing concentrations of SS for up to 72 h. Cell viability was detected by MTT assay. The operational approach has been reported in a previous study (32). Lastly, an ELISA reader (Perkin Elmer, Victor X5, Waltham, MA, USA) was used to measure the absorbance at 570 nm. The calculation formula of cell viability (%) was as follows: (absorbance of test sample/absorbance of control) × 100. The cells treated with vehicle only (DMSO, 0.1% in media) was served as a zero control and the control values were set to 1 by default.

### EdU Incorporation Assay

HepG2 and QGY-7703 cells (5 x 10^3^ cells/well) were seeded into 96-well plates followed by treating with SS (45 μM) for 24 h. After 24 h, the medium was removed and the cells was cultured in a resuspended RPMI-1640 medium with 50 μM EdU for 2 h at 37°C, stained with Apollo reaction reagent. All DNA contents of the cells were stained with Hoechst 33342. At last, an inverted fluorescence microscope (Nikon, Ts2RFL, Tokyo, Japan) was used to take pictures at × 400 magnifications. Three captured fields were selected randomly and the EdU-positive cells were calculated. The calculation formula was as follows: percentage of EdU-positive cells = (EdU-positive cells/Hoechst stain cells) × 100.

### Quantitative Real-time PCR

Total RNA of HepG2 and QGY-7703 cells from different treatment were extracted by using Trizol reagent (Invitrogen). RNA was reversed transcribed into cDNAs using the RT-PCR kit (TaKaRa, Dalian, China). The reverse-transcription step was carried out in triplicate and the total RNA concentration was the same in every sample. A quantitative real-time RT-PCR (qRT-PCR) assay was performed on an ABI 7500 Real-Time PCR System (Applied Biosystems, Grand Island, NY, USA) for the quantification of miR-375-3p and CCAT1 transcript using the SYBR Premix Dimmer Eraser kit (TaKaRa) and fluorescent RNA-binding dyes. All conductions were in accordance with the instructions provided by the manufacturer. Each sample was tested in triplicate, and reference genes were applied to normalize the results. The PCR conditions were as follows: 10 min at 95°C, followed by 40 cycles of 15 s at 95°C, and 1 min at 60°C. Threshold quantification cycle *(C*_*q*_*)* was determined for each sample/primer pair and the 2–ΔΔCt method was used to calculate the relative levels of specific molecules. The copy numbers were consistent with the anticipated result. The amplification efficiency for miR-375-3p and CCAT1 was 100.95 and 100.35%, respectively. The forward and reverse primer sequences used in qRT-PCR are shown in Table 2 (Supplementary Material). The procedure was based on the guidelines of the minimum information for publication of qRT-PCR experiments (MIQE) (33).

**Table 2:** The primer sequences of gene amplification by qRT-PCR.

**Symbol**	**Primer**	**Primer sequence(5^**′**^-3^**′**^)**
CCAT1	F-primer	5′-GCCGTGTTAAGCATTGCGAA-3′
	R-primer	5′-TCATGTCTCGGCACCTTTCC-3′
GAPDH	F-primer	5′-AAGCCTGCCGGTGACTAAC-3′
	R-primer	5′-GCGCCCAATACGACCAAATC-3′
miR-375-3p	F-primer	5′-TGCTTTGTTCGTTCGGCTC-3′
	R-primer	5′-TATGGTTGTTCACGACTCCTTCAC-3′
U6	F-primer	5′-ATTGGAACGATACAGAGAAGATT-3′
	R-primer	5′-GGAACGCTTCACGAATTTG-3′

### Western Blot Analysis

HepG2 and QGY-7703 cells were seeded into 6-well plates at a density of 4 × 10^5^ cells/well or 2 × 10^5^ cells/well and treated with different conditions of SS for up to 48 h. The cells were lysed with 1 × RIPA buffer, which contained proteinase inhibitor cocktail, and the protein concentrations were measured. Equal amounts of protein were mixed in volumetric 3 × SDS sample buffer and separated on 10% SDS polyacrylamide gels. Primary antibody was incubated at 4°C overnight. Afterwards, secondary antibody raised against rabbit IgG conjugated to horse-radish peroxidase (Cell Signaling, Beverly, MA, USA) was incubated for 1 h at room temperature. Finally, signals were detected using a freshly prepared enhanced chemiluminescence solution (Millipore, Burlington, MA, USA) with a ChemiDoc XRS +System (Bio-Rad, Hercules, CA, USA). ImageJ software (National Institutes of Health, Bethesda, MD, USA) was used to quantify and compare the intensity of single band between the control and proteins of interest.

### Transient Transfection Assays

HepG2 and QGY-7703 cells (2 × 10^5^ cells/well) were seeded into 6-well plates and reached to 50–60% confluence before treatment. The mimics, inhibitors and the negative control of miR-375-3p were mixed with the ribo FECT™ CP transfection reagent (RiboBio Co., Guangzhou, China) in accordance with the instructions provided by the manufacturer; compounds were added to the cells and maintained for 48 h at 37°C. In separate experiments, HepG2 and QGY-7703 cells were seeded into 6-well plates at a density of 3 × 10^5^ cells/well and transfected with the pcDNA3.1 (control plasmid), pcDNA3.1-SP1, overexpression plasmids of CCAT1 and IRF5 (EX-NEG-M02-CCAT1, EX-NEG-M02-IRF5), and the respective controls obtained from GeneCopoeia, Inc. (Rockville, MD, USA) with Lipofectamine 3000 reagent at a final concentration of 2 μg/mL for 6 h at 37°C followed by treatment with SS for the indicated time for all other experiments.

### Luciferase Reporter Assay

HepG2 and QGY-7703 cells were seeded into 24-well plates at a density of 6.5 × 10^4^ cells/well and reached to 50–60% confluence before treatment. The control plasmid pEZX-PL01 and pEZX-PL01-IRF5 promoter plasmids purchased from GeneCopoeia (Rockville, MD, USA) were transfected into the cells with Lipofectamine 3000 for 6 h, followed by treating with SS for an additional 24 h. The wild and mutation types of CCAT1 3′-UTR luciferase vectors were designed and synthesized by GeneCopoeia, Inc. (Rockville, MD, USA). These vectors were co-transfected into the cells with either miR-375-3p mimic or a negative control using Lipofectamine 3000 Reagent, followed by exposure of the cells to SS for an additional 24 h. The preparation of cell lysis and the measurement of luciferase activities were determined using the Luc-Pair™ Dual-Luminescence Assay Kit (GeneCopoeia), in accordance with the instructions provided by the manufacturer. In a separate experiment, the control and IRF5 promoter were transfected into the cells for 6 h before transfecting with the pcDNA3.1 and SP1 overexpression plasmids, and treated with SS for 24 h, followed by measuring luciferase activity using the Luc-Pair™ Dual-Luminescence Assay Kit (GeneCopoeia).

### RNA Immunoprecipitation (RIP) Assay

The RNA immunoprecipitation (RIP) assay was performed using the Magna RIP™ RNA-Binding Protein Immunoprecipitation Kit (Millipore, Billerica, MA, USA), according to the manufacturer's instructions. Briefly, HepG2 and QGY-7703 cells (2.0 × 10^7^) were rinsed and scraped with cold PBS, then lysed in complete RIP lysis buffer containing protease and RNase inhibitors. The cell lysis was incubated with RIP immunoprecipitation buffer containing magnetic beads conjugated with human anti-Ago2 antibody (Millipore, Billerica, MA, USA) (5 μg of total antibody used per immunoprecipitation) at room temperature for 30 min, and negative control IgG (Millipore, Billerica, MA, USA). The beads were then thoroughly washed and digested with proteinase K (30 min at 55°C) to disengage Ago2, containing ribonucleoprotein (RNP) complexes. Purified RNA was obtained and then applied to quantitative PCR with reverse transcription analysis. The expression of Ago2 was measured by Western blot.

### Xenograft Tumor Study

Female nude mice (weight of 18–20 g), which were purchased from Beijing Vital River Laboratory Animal Technology Co., Ltd. (Beijing, China), were kept in a SPF environment at the Animal Center of Guangdong Provincial Hospital of Chinese Medicine. All animal experimental procedures were performed in accordance with the protocol approved by the Animal Care and Use Committee of Guangdong Provincial Hospital of Chinese Medicine and the National Institutes of Health Guide for the Care and Use of Laboratory Animals (the Ethics Approval Number 2018067). HepG2-Luc cells carrying luciferase reporter gene (HepG2-Luc, obtained from the Guangzhou Land Biological Technology Co., Guangzhou, China) were resuspended in 0.2 mL of phenol red-free RIPM 1640 with 2% FBS in a number of 2.0 × 10^6^. The resuspended cells were then injected into the upper hind limb of the nude mice. Xenografts were expected to grow for 1 week when starting the first measurements. Mice were randomly divided into three groups: the control, low-dose group (SS, 5 mg/kg), and high-dose group (SS, 20 mg/kg), and were injected with reference substance or SS once a day via intraperitoneal injection for up to 15 days (*n* = 9 per group). Mice were then anesthetized by inhalation of 2% isoflurane and injected with the substrate D-Luciferin (Caliper Life Sciences, Hopkinton, MA, USA) at a dose of 150 mg/kg in 100 μL into the peritoneal cavity of the nude mice. The bioluminescence imaging signal was determined using the IVIS200 Imaging System (Xenogen/Caliper, Alameda, CA, USA) at the first and end of the experiments (on day 2 and 15) and expressed as photons/sec. Tumor volume was calculated using the formula for a spheroid: volume = (width^2^ × length) × 0.5 and the mice weights were measured once a week. All mice were sacrificed on the 15th day in accordance with the Guidelines for the Care and Use of Laboratory Animals. At the end of the experiments, xenograft tumors were isolated and expressions of miR-214-3p, CCAT1, SP1, and IRF5 were determined by qRT-PCR and Western blot, respectively.

### Statistical Analysis

Data were generated from at least three separated experiments. Continuous variable of the date are presented as the mean ± SD. One-way ANOVA was used to detect the differences between groups, and significance of difference between particular treatment groups was analyzed by Dunnett's multiple comparison tests. GraphPad Prism software was used to create the diagram, and asterisks which indicated *P* < 0.05, showed the significant differences between experimental groups and the corresponding control condition.

## Results

### Solasonine (SS) Inhibited Growth of HCC Cells

**Figure 1:**
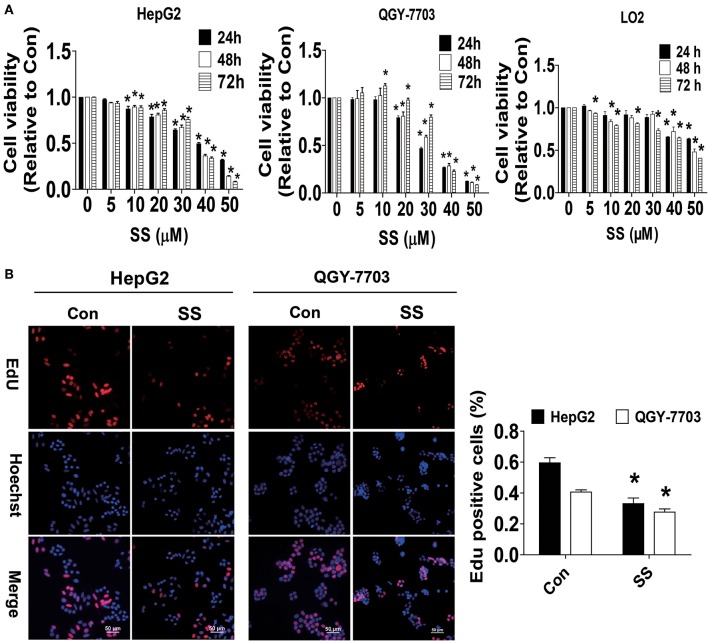
SS inhibited growth of HCC cells. **(A)** HCC HepG2 and QGY-7703 cells (3–5 x 10^3^ cells/well), normal hepatocyte (LO2) cells (3–5 x 10^3^ cells/well) were treated with different concentrations of SS for up to 72 h. The cells were collected and processed for MTT assay as described in the Materials and Methods section. **(B)** HepG2 and QGY7703 cells were treated with SS (45 μM) for 24 h, followed by processing for measuring the cell growth by EdU DNA cell proliferation kit described in the Materials and Methods section. The image was magnified 10×. Hoechst was used to stain all the nuclei. At least 5 captured fields were randomly selected, and the percentage of EdU positive cells = (EdU positive cells/Hoechst stain cells) × 100. Scale bars, 50 μM. Values are given as the mean ± SD, from three independent experiments performed in triplicate. ^*^Indicates significant difference as compared to the untreated control group (*P* < 0.05).

Previous studies from ours and others have shown that bioactive glycoalkaloids, such as SS, solasodine, and solamargine, inhibited growth of different cancer cells (8, 34, 35). In the current study, we showed that SS inhibited the growth of HCC cells in time-and dose-dependent manner as determined by MTT assay (Figure 1A). The IC50 values were 37.70, 33.88, 35.48 μM and 29.17, 31.83, 35.01 μM from 24 to 72 h in HepG2 and QGY7703 cells, respectively. This finding was also proven by another method for detecting cell proliferation-EdU incorporation assay. Please note that much lower toxicity profiles were observed when human hepatocytes LO2 cells were exposed to the same concentration of SS (IC50 values were 62.43, 49.84, 51.91 μM) from 24 to 72 h (Figure 1A). We demonstrated that the percentage of EdU positive HCC cells was significantly reduced in the SS-treated group compared to the control one (Figure 1B). These results suggested that SS inhibited the growth of HCC HepG2 and QGY7703 cells.

### SS Increased the Expression of miR-375-3p and Inhibited the Levels of lncRNA CCAT1, and There Was Reciprocal Interaction of CCAT1 and miR-375-3p in HCC Cells

**Figure 2:**
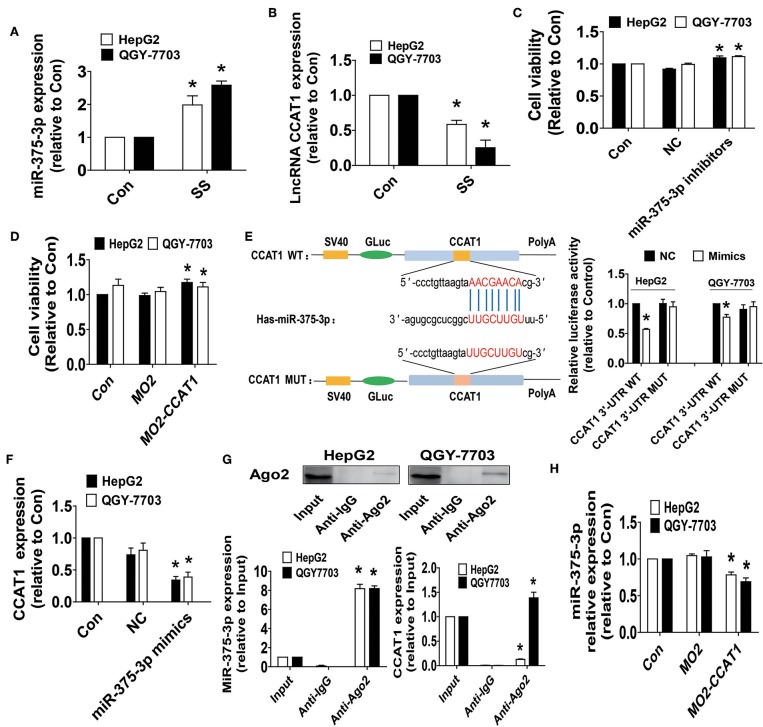
SS increased the expression of miR-375-3p and inhibited the levels of lncRNA CCAT1, and there was reciprocal interaction of CCAT1 and miR-375-3p in HCC cells. **(A,B)** HepG2 and QGY7703 cells were treated with SS (45 μM) for 24 h, and the expression levels of miR-375-3p and CCAT1 were measured via qRT-PCR. **(C,D)** HepG2 and QGY7703 cells were transfected with the control or miR-375-3p mimics (100 nM), CCAT1 expression vectors for up to 48 h followed by determining cell growth via MTT assays. **(E)** The luciferase reporter constructs containing the wild type and mutant binding sites in 3′-UTR region of CCAT1 were shown (upper panel). HepG2 and QGY7703 cells were transfected with the CCAT1 3'UTR-WT or CCAT1 3′-UTR-Mut vectors (1.25 μg/mL each) for 24 h, then treated with the miR-375-3p mimics (100 nM) or miR-negative control (NC) for an additional 48 h. Afterwards, the luciferase activity was detected using Secrete-Pair™ Dual Luminescence Assay Kit as described in the Materials and Methods section (lower panel). **(F)** HepG2 and QGY7703 cells were treated with the control or miR-375-3p mimics (100 nM) for up to 48 h followed by determining the expression levels of CCAT1 via qRT-PCR. **(G)** Cell lysates from HepG2 and QGY7703 cells were incubated with Ago2 antibody-coated magnetic beads. Precipitates ware subjected to Western blot for Ago2 protein and qRT-PCR for detecting CCAT1 and miR-375-3p expression levels. Preimmune IgG and input from cell extracts were used as controls. **(H)** HepG2 and QGY7703 cells were transfected with the control or CCAT1 overexprssion vectors for up to 48 h followed by determining the expression levels of miR-375-3p via qRT-PCR. Values in bar graphs were given as the mean ± SD from three independent experiments. ^*^Indicates significant difference as compared to the untreated control group (*P* < 0.05).

We next examined the possible targets that may be involved in the inhibitory effect of SS on cell growth. Studies have demonstrated the important roles of lncRNAs and miRNA, such as CCAT1 and miR-375, in different types of cancers, including HCC, and aberrant expressions of CCAT1 and miR-375 have been involved in several biological processes, such as cell proliferation, migration, and invasion, via regulating different target genes and signaling pathways (36–40). However, the biological role of either CCAT1 or miR-375 in HCC remains to be incompletely characterized. It was for these reasons that we explored the role of CCAT1 and miR-375 in mediating the anti-HCC effect of SS. Herein, our results unveiled that SS significantly increased miR-375-3p, while reduced lncRNA CCAT1 expression levels were observed in HepG2 and QGY7703 cells (Figures 2A,B). Of note, either the inhibitors of miR-375-3p or exogenously expressed CCAT1 significantly stimulated the growth of HepG2 and QGY7703 cells as determined by MTT assay (Figures 2C,D). Bioinformatics analyses have found that miR-375-3p could physical bind to CCAT1, we therefore want to examine whether miR-375-3p regulated expression of CCAT1. We found that the mimics of miR-375-3p reduced the luciferase activity in 3-UTR region of CCAT1 in HepG2 and QGY7703 cells (Figure 2E), and suppressed the expression of CCAT1 (Figure 2F). In addition, AGO_2_ RIP assays showed that CCAT1 could bind with miR-375-3p (Figure 2G). Interestingly, we showed that exogenously expressed CCAT1 reduced the expression of miR-375-3p in HepG2 and QGY7703 cells (Figure 2H). Together, our results demonstrated that CCAT1 was a target of miR-375-3p and there was a reciprocal interaction between CCAT1 and miR-375-3p, which may be important targets of SS. Furthermore, inhibition of CCAT1 and induction of miR-375-3p were involved in the SS-mediated inhibition of HepG2 and QGY7703 cell growth.

### SS and the Mimics of miR-375-3p Reduced SP1 Protein Expression Whereas Overexpressed CCAT1 Enhanced SP1 Protein Expression

**Figure 3:**
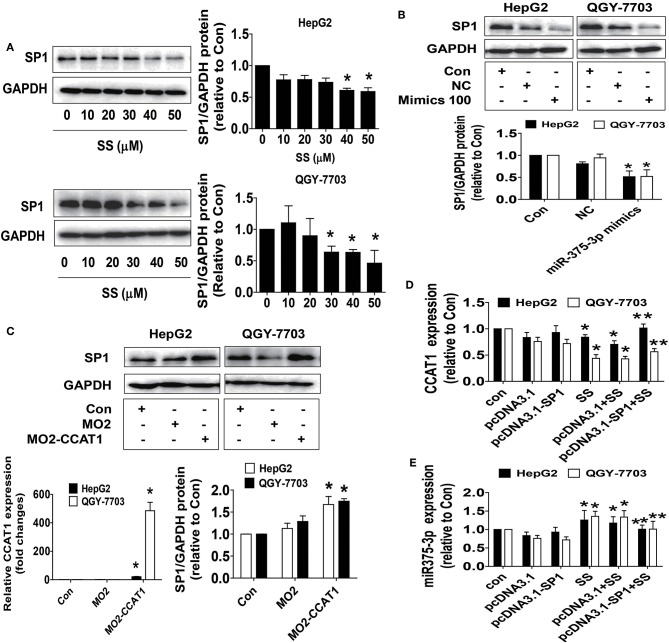
SS and the mimics of miR-375-3p reduced SP1 protein expression whereas overexpressed CCAT1 enhanced SP1 protein expression. **(A)** HepG2 and QGY7703 cells were treated with different concentrations of SS for 24 h. The expression of SP1 protein was detected by Western blot. GAPDH was used as a loading control. **(B,C)** HepG2 and QGY7703 cells were treated with mimics of miR-375-3p or transfected with the control and excessive expressed CCAT1 vector for up to 24 h before exposing the cells to SS (45 μM) for an additional 24 h. Afterwards, the expressions of SP1 proteins were detected by Western blot. GAPDH was used as a loading control. The figures are representative cropped gels/blots that have been run under the same experimental conditions. **(D,E)** HepG2 and QGY7703 cells were transfected with the control or SP1 expression vectors for 24 h before exposing the cells to SS (45 μM) for an additional 24 h followed by measuring the expression levels of CCAT1 and miR-375-3p via qRT-PCR. Values in bar graphs were given as the mean ± SD from three independent experiments. ^*^Indicates significant difference as compared to the untreated control group (*P* < 0.05); ^**^Indicates significant difference from SS treated alone (*P* < 0.05).

To investigate the mechanism underlying the SS-regulated CCAT1 and miR-375-3p expressions, and identify relevant downstream target, we next began to test the biological significance of the interaction of CCAT1 and miR-375-3p in mediating the effect of SS. Transcription factors, such as SP1, have been shown to regulate the expression of multiple genes implicated in several biological functions, such as cell proliferation, progression, and cell death (41). More importantly, bioinformatics analysis and other experimental procedures showed that SP1 was a direct target of miR-375-3p (42–44). We found that SS reduced SP1 protein expressions (Figure 3A). Moreover, the mimics of miR-375-3p reduced, whereas excessive expression of CCAT1 enhanced SP1 protein expression in HepG2 and QGY7703 cells (Figures 3B,C). Interestingly, exogenously expressed SP1 was found to feedback resist SS-inhibited CCTA1 expression (Figure 3D), and SS-stimulated miR-375-3p (Figure 3E). These findings indicated that both CCAT1 and miR-375-3p acted as upstream factors, regulated the expression of SP1, and there were feedback regulatory loops between CCAT1, miR-375-3p and SP1, leading to the reciprocal interactive axis in this process.

### SS Reduced IRF-5 Protein Expressions and Promoter Activity, Which Were Reversed by Excessive Expressed SP1

**Figure 4:**
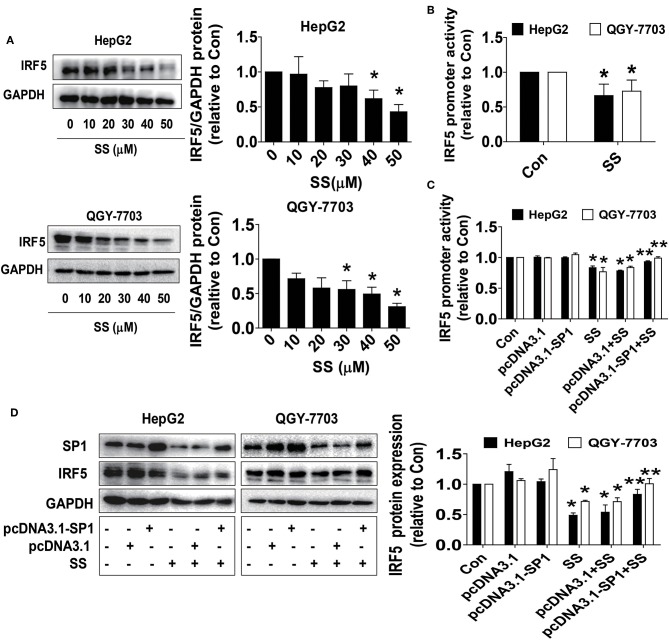
SS reduced IRF-5 protein expressions and excessive expressed IRF5 neutralized SS-inhibited cell growth. **(A,B)** HepG2 and QGY7703 cells were treated with different concentrations of SS for 24 h or a wild type human IRF5 promoter reporter construct ligated to luciferase reporter gene and the internal control for 24 h, followed by treating with the SS (45 μM) for an additional 24 h. The expression of IRF5 protein and promoter activities were determined by Western Blot **(A)** and Dual-Luciferase Reporter Assay System **(B)** described in the Materials and Methods section. The figures are representative cropped gels/blots that have been run under the same experimental conditions. **(C,D)** HepG2 and QGY7703 cells were transfected with the control or SP1 expression vectors for 24 h, or a wild type human IRF5 promoter reporter construct ligated to luciferase reporter gene and the internal control for 24 h, followed by treating with the SS (45 μM) for an additional 24 h. Afterwards, The expression of IRF5 protein and promoter activities were determined by Dual-Luciferase Reporter Assay System **(C)** and Western Blot **(D)** and described in the Materials and Methods section. The figures are representative cropped gels/blots that have been run under the same experimental conditions. Values in bar graphs were given as the mean ± SD from three independent experiments. ^*^Indicates significant difference as compared to the untreated control group (*P* < 0.05); ^**^Indicates significant difference from SS treated alone (*P* < 0.05).

The master transcription factor IRF5 has been involved in the occurrence and progression of numerous diseases, including cancer (45). Bioinformatics analyses showed that the IRF5 promoter region contained putative SP1 binding sites, and a series of gain and off-functional experiments suggested that the SP1 transcription factor was the primary determinant for activating the basal transcription of the IRF5 (46). Herein, we further delineate the association and role of IRF5 in this process. We showed that SS inhibited IRF5 protein expression in a dose-dependent fashion (Figure 4A) and the promoter activity in HepG2 and QGY7703 cells (Figure 4B), which was overcome in cells overexpressed SP1 gene in HepG2 and QGY7703 cells (Figures 4C,D). These findings confirmed that SP1, which acts as upstream factor of IRF5, regulated the expression of IRF5 in this process.

### IRF5 Feedback Regulated CCAT1 Expression and Neutralized SS-Inhibited Cell Growth

**Figure 5:**
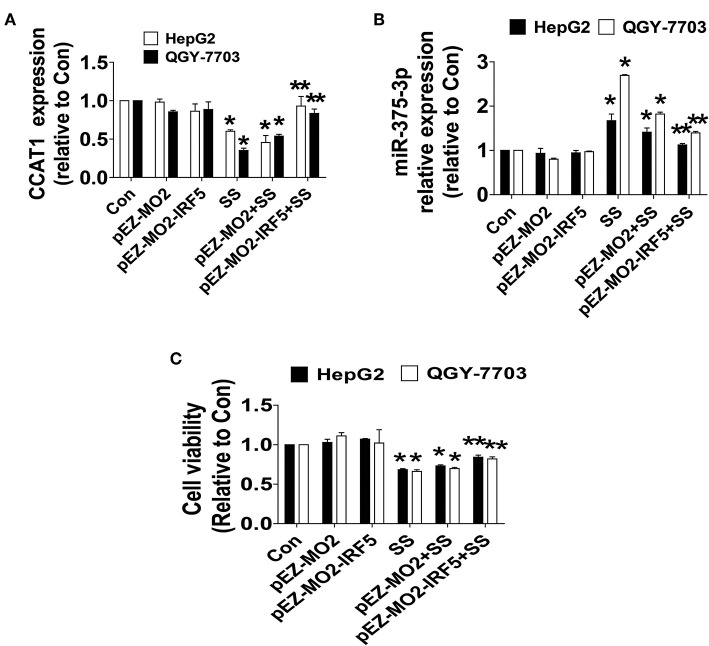
IRF5 feedback regulated CCAT1 and miR-375-3p expression and neutralized SS-inhibited cell growth. **(A–C)** HepG2 and QGY7703 cells were transfected with the control or IRF5 expression vectors for 24 h followed by treating with the SS (45 μM) for an additional 24 h. Afterwards, The expression of CCAT1 and miR-375-3p, and cell growth were determined by qRT-PCR and MTT described in the Materials and Methods section, respectively. Values in bar graphs were given as the mean ± SD from three independent experiments. ^*^Indicates significant difference as compared to the untreated control group (*P* < 0.05); ^**^Indicates significant difference from SS treated alone (*P* < 0.05).

To further delineate the role and illustrate the function of IRF5 in HCC growth, we assess the possibility of feedback regulatory loops. We showed that exogenously expressed IRF5 unexpectedly antagonized the SS-inhibited CCAT1, and SS-induced miR-375-3p expressions (Figures 5A,B), and more importantly, neutralized SS-inhibited HCC cell growth (Figure 5C). Three findings indicated that there were feedback regulatory loops and IRF5 played a critical role in mediating the SS-inhibited HCC cell proliferation.

### The Anti-HCC Effects by SS in a Mouse Xenograft Tumor Model

**Figure 6:**
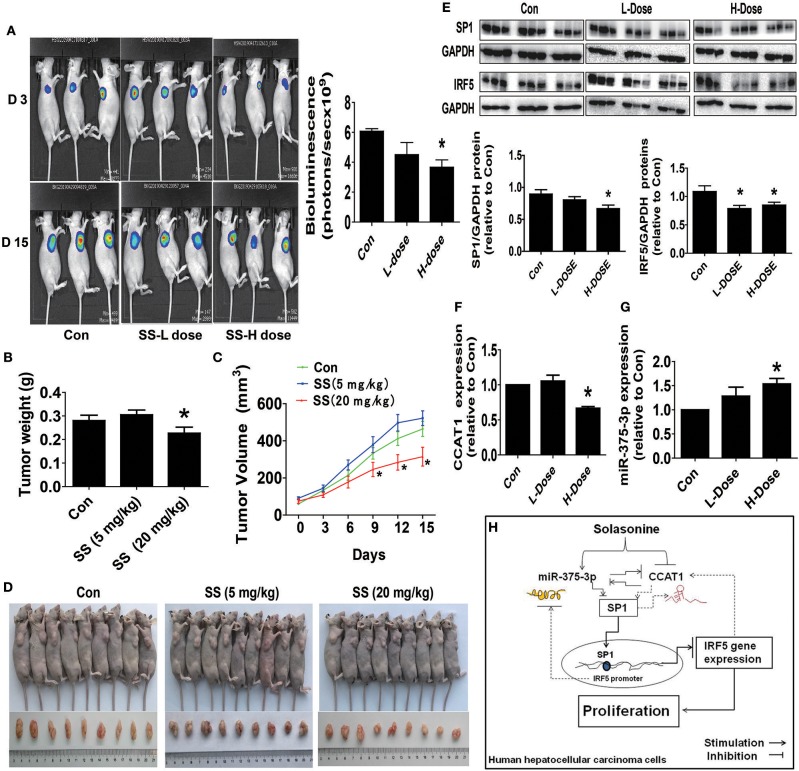
The anti-HCC effects by SS in a mouse xenograft tumor model. **(A)** HepG2-Luc cells carrying luciferase reporter gene (HepG2-Luc, obtained from the Guangzhou Land Biological Technology Co., Guangzhou, China) were resuspended in 0.2 mL of phenol red-free RIPM 1640 with 2% FBS in a number of 2.0 × 10^6^. Then, the resuspended cells were injected into the upper hind limb of the nude mice. Xenografts were expected to grow for 1 week when starting the first measurements. Mice were randomly divided into three groups: the control, low-dose group (SS, 5 mg/kg), and high-dose group (SS, 20 mg/kg), and were injected with reference substance or SS once a day via intraperitoneal injection for up to 15 days (*n* = 9 per group). The xenografts were assessed by *in vivo* bioluminescence imaging at the first and end of the experiments (on day 2 and 15). The tumor growth was monitored by injecting luciferin in the mice followed by measuring bioluminescence using IVIS Imaging System. Imaging and quantification of signals were controlled by the acquisition and analysis software living image as described in the Materials and Methods section. Representative images are shown. **(B,C)** The xenografts were harvested on day 15, and the weight **(B)** and volume **(C)** of tumors were measured. **(D)** The photographs of the vehicle- and drugs-treated xenografts derived from nude mice are shown. **(E–G)** At the end of the experiments, xenograft tumors were isolated from individual animals, and the corresponding lysates were processed and detected miR-375-3p and CCAT1 levels, SP1 and IRF5 protein expressions by qRT-PCR and Western blot, respectively. GAPDH was used as a loading control. The figures are representative cropped gels/blots that have been run under the same experimental conditions. The bar graphs represented the tumor weight and volume of mice results of as mean ± SD. ^*^Indicates the significant difference from the untreated control (*p* < 0.05). **(H)** The diagram shows that SS inhibits HCC growth through the reciprocal regulation between the miR-375-3p and lncRNA CCAT1, this result in transcription factor SP1-mediated reduction of IRF5 gene expression. The interactions among miR-375-3p, CCAT1, SP1, and IRF5 axis unveil a novel molecular mechanism underlying the anti-HCC growth by SS.

Finally, we further examined the role of SS on tumor growth *in vivo*. Mice bearing xenografted HCC HepG2-Luc cells were treated via intraperitoneal injection with either the control or SS for up to 15 days, followed by being given D-luciferin via intraperitoneal injection. The xenografts were assessed by *in vivo* bioluminescence imaging at the start and end of the experiments (on day 2 and 15). Through the Xenogen IVIS200 system, we found that the high doses of (20 mg/kg) SS-treated mice had a substantial inhibitory effect on tumor growth as compared to that in the control group (Figure 6A). In addition, compared to that in the control group, a significant reduction in the xenografted tumor weight and size (volume) was observed in the high dose of SS-treated group (Figures 6B–D). Moreover, consistent with the results from the *in vitro*, we observed the induction of miR-375-3p and reductions of CCAT1 expressions and SP1 and IRF5 protein levels from xenografted tumors obtained from the above experiments in the high dose SS-treated group, as compared to that in the control one, as determined by qRT-PCR and Western Blot, respectively (Figures 6E–G).

## Discussion

Several natural compounds and phytochemicals demonstrated many biological activities, such as antibacterial and anticancer (47). Previous studies from ourselves and others have shown that bioactive glycoalkaloids, such as SS, solasodine, and solamargine, inhibited growth of several different cancer cells (8, 34, 35). These results suggested the therapeutic potential of SS in cancer treatment. However, the underlying molecular mechanisms in controlling human cancer proliferation by this agent still need to be determined. In the current study, we provided new evidence demonstrating the anti-HCC effect of SS. We observed that SS inhibited growth of HCC HepG2 cells through the reciprocal regulation between the miR-375-3p and lncRNA CCAT1, and this resulted in transcription factor SP1-mediated reduction of IRF5 gene expression. Our results showed relatively high IC50 values of SS in HepG2 and QGY7703 cells, respectively. We believe that the cell culturing and growth conditions when cells are exposed to SS, the genetic and biological variation of the HCC cell line itself and other unknown factors may have contributed to this relatively high dose response, although this may be the result of physiological ranges without noticeable toxicity. Consistent with this, one recent study showed that SS had cytotoxicity on HepG2 cells with IC50 of 91.8 ± 9.4 μM, while solamargine showed comparable potency against HepG2 cells with IC50 of 10.8 ± 0.1 μM, suggesting relatively weak cytotoxicity of SS in HCC cells (34). Of note, low IC50 value was reported in other cancer cell types using SS (5). Regardless, more experiments are strongly required to confirm potential anti-proliferative/cytotoxic effects of SS against cancer.

In this study, we demonstrated a role of miR-375-3p and lncRNA CCAT1 in mediating the anti-HCC cell growth. Our results indicated that the induction of miR-375-3p and reduction of CCAT1 involved in the SS-inhibited growth of HCC HepG2 cells and QGY7703 cells. MiR-375, acting as tumor suppressor, significantly inhibited cell proliferation and induced apoptosis in cancer cells via different mechanisms (36, 48, 49). However, the role of miR-375 in HCC has not been reported. Consistent with this, our results confirmed the tumor suppressor role and suggested the involvement of this miRNA in mediating the anti-HCC cell growth by SS. Our results also implied the oncogenic role of CCAT1 in this study. Studied have shown that CCAT1 played important roles in many cancers by stimulating cell proliferation, migration, invasion and metastasis (50). Interestingly, we demonstrated a reciprocal regulation between the miR-375-3p and CCAT1 in mediating the SS effect in this process suggesting that there is direct binding of miR-375-3p to CCAT1, and CCAT1 was a direct target of miR-375-3p. The interaction of CCAT1 with miRNAs, other than miR-375, have been largely reported in other studies in different cancer types (38, 51, 52). CCAT1 was highly expressed in HCC tissues and cells, and involved in growth and metastasis. CCAT1 stimulated proliferation of HCC cells via regulation of CCNE1 expression by acting as a ceRNA to sponge miR-30c-2-3p (38). Our findings suggested that the CCAT1/miR-375-3p regulatory axis could be a potential target for HCC treatment. Other regulatory axes, such as CCAT1/let-7/high mobility group A2 (HMGA2) and c-Myc, have been reported to be involved in the HCC growth and invasion and metastasis (11). We believed that this would add the significant role of CCAT1, and implicate the potential application of CCAT1 for the prognosis and treatment of HCC. These also suggest that multiple targets and regulatory pathways have been involved in the anti-HCC effects. More importantly, we have observed how CCAT1 acted as ceRNA to sponge miR-375-3p, and there was a physical binding of CCAT1 to miR-375-3p affected in the presence of SM, resulting in the inhibition of CCAT1 expression. The true significance of this association and detailed mechanism underlying this process still needs to be determined in the future research.

We observed the role of transcription factor SP1. Our results suggested that both CCAT1 and miR-375-3p acted as upstream factors, regulating the expression of SP1 in this process. As a common transcription factor, SP1 has been associated with many biological processes, such as growth, apoptosis, metastasis, drug resistance, differentiation, DNA damage response and angiogenesis (41, 53, 54). Studies using bioinformatics analysis and other experimental procedures, such as 3′-UTR luciferase activity assays, have confirmed that SP1 is a target of miR-375-3p (42–44). Consistent with this, our results suggested the oncogenic role of SP1 in mediating the anti-HCC effect of SS. We also demonstrated the feedback role of SP1 on CCAT1 and miR-375-3p expressions, suggesting a potential complex regulatory loops, which needs to be determined in the future. As a critical transcription factor, IRF5 regulated immune and inflammatory responses in host defense and disease (55). Studies also showed the role of this transcription factor in cancer biology (56–58). IRF5 expression and function in hepatocytes infected with HCV virus, HCV replicon cells, and human primary tissues from patients with HCV-positive and -negative HCC were examined and identified that IRF5 was a new negative regulator of HCV-associated HCC pathogenesis. IRF5 induced apoptosis, inhibited autophagy, and suppressed migration, invasion of hepatocytes infected with HCV virus and HCV replicon cells. Thus, IRF5 acted as an important suppressor of HCV replication and HCC pathogenesis (45). On the contrary, IRF5 played an adverse role in predicting both OS and RFS in patients with non-metastatic ccRCC (27). Although limited data demonstrated the dual role of IRF5, our results suggested that repression of IRF5 contributed to the overall effect of SS in HCC inhibition. More specifically, our results indicated that the inhibition of HCC by SS was partly due to the observation that SP1 could bind to the promoter regions of IRF5, thereby directly regulating the expression of the IRF5 gene. Consistent with these findings, one study demonstrated the association between SP1 and IRF5. Bioinformatic analyses showed that the promoter region of IRF5 contained several putative SP1 binding sites. Excessive expression of SP1 enhanced the promoter activity and increased the expression of IRF5, suggesting that SP1 transcription factor is the primary positive determinant for increasing the expression of IRF5 (46). Overall, our findings demonstrated that the regulation, interplay and potential regulatory mechanisms among CCAT1 and miR-375-3p, and SP1 and IRF5, converge in the anti-HCC effect of SS. More studies are still required to further elucidate the in-depth mechanism underlying these correlations that contributed to overall effect of SS in HCC growth inhibition.

Moreover, our *in vivo* results were consistent with the findings *in vitro*, confirming the suppressive effects of SS on HCC HepG2 tumor growth and regulations of CCAT1, miR-375-3p, SP1, and IRF5 expressions. The doses of SS used were based on previous studies (8, 59), which demonstrated remarkable inhibitory effects without noticeable toxicities. Our findings suggested that SS suppressed growth of human HCC HepG2 cells, via targeting CCAT1/miR-375-3p/SP1/IRF5 signaling regulatory axis.

Of note, one major limitation in this study was that the only true HepG2 HCC cell line was used, as the previously considered HCC cell line, QGY-7703, was recently identified to be an unreliable cell line model for HCC due to the potential contamination of other human cell lines. Although similar results were also obtained from this cell lines in the current study. We believe that using other reliable HCC cell lines, such as Hep3B, HCCLM3, and MHCC-97H, is required to confirm our findings.

In summary, our results show that SS inhibited growth of HepG2 HCC and QGY-7703 cells through the reciprocal regulation between the miR-375-3p and lncRNA CCAT1, which leads to transcription factor SP1-mediated reduction of IRF5 expression (Figure 6H). The interactions and inter-regulations among miR-375-3p, CCAT1, SP1, and IRF5 axis unveil a novel molecular mechanism underlying the anti-HCC growth by SS. IRF5 may be a potential target for HCC therapy. Additional experiments using other reliable cell line models for HCC are strongly desirable to support the conclusion. Moreover, the available data of correlations among IRF5, CCAT1, and miR-375 and HCC patient survival were scarce and the public datasets had little such information thus far, although the differential expressions of these molecules between HCC tumor and normal tissues have been shown and associated with the prognosis, and patient survival (27), and have acted as potential biomarkers in diagnosis and prognosis of HCC (11, 12, 60). Regardless, future, well-designed, large-size, and high quality patient cohort studies are required to elucidate the clinic-pathological implications of IRF5, CCAT1, and miR-375 in patients with HCC. For example, overall survival using GEPIA (Gene Expression Profiling Interactive Analysis) web server (http://gepia2.cancer-pku.cn/) is a valuable resource for gene expression analysis based on tumor and normal samples from The Cancer Genome Atlas (TCGA) and Genotype-Tissue Expression (GTEx) datasets, and will be one of the most preferred tools for biologists and clinicians to explore cancer genomics data (61).

## Data Availability Statement

The raw data supporting the conclusions of this manuscript will be made available by the authors that uploaded in the supplementary section to any qualified researcher.

## Ethics Statement

All animal experimental procedures were performed in accordance with the protocol approved by the Animal Care and Use Committee of Guangdong Provincial Hospital of Chinese Medicine and the National Institutes of Health Guide for the Care and Use of Laboratory Animals (the Ethics Approval Number 2018067).

## Author Contributions

SH conceived of the study, participated in its design and coordination, draft and finalized the manuscript. ZL, CM, and XT carried out the cell growth, siRNA, Western Blot assays, transfection and luciferase reporter assays, and statistical analysis. QT, FZ, and LL participated in performed the cell viability, siRNA, transfection assays, and protein expression experiments. YY and JW performed statistical analysis. XY and WW coordinated and provided important suggestions including some reagents, and critical reading the manuscript. All authors read and approved the final manuscript.

### Conflict of Interest

The authors declare that the research was conducted in the absence of any commercial or financial relationships that could be construed as a potential conflict of interest.
