# Supplementary material for: The Reciprocal Interaction Between LncRNA CCAT1 and miR-375-3p Contribute to the Downregulation of IRF5 Gene Expression by Solasonine in HepG2 Human Hepatocellular Carcinoma Cells
Source: Front Oncol. 2019 Oct 18;9:1081. doi: 10.3389/fonc.2019.01081 (PMC6813207; doi:10.3389/fonc.2019.01081)
Supplement: Supplementary file 1 [file Data_Sheet_1.PDF]

The reciprocal interaction between LncRNA CCAT1 and miR-375-3p contribute to the downregulation of IRF5 gene expression by solasonine in human hepatocellular carcinoma cells

Zheng Liu[1], ChangJu Ma[1], XiaoJun Tang[1], Qing Tang[1], LiJie Lou[2], Yaya Yu[1], Fang Zheng[1], JingJing Wu[1], Xiao-bo Yang[3*], Wei Wang[2, 3*], Swei Sunny Hann[1, 3*]

[1]Laboratory of Tumor Biology, [2]Department of Gastrointestinal Surgery, [3]Guangdong Provincial Key Laboratory of Clinical Research on Traditional Chinese Medicine Syndrome, The Second Clinical College of Guangzhou University of Chinese Medicine, Guangzhou University of Chinese Medicine, Guangzhou, Guangdong Province, China, 510120

Running title: Repression of IRF5 gene expression by solasonine

[*]Address all correspondence to
Swei Sunny Hann, MD, PhD, Xiao-bo Yang, MD, PhD, Wei Wang, MD, FRCP
No. 111, Dade Road, Guangzhou, Guangdong Province, P. R. China, 510120
Tel: 020-39318472
Email: hann20102@outlook.com, yangxiaobomd@163.com, ww1640@yeah.net

Fig 1 A

HepG2 — OD value(570 nM)

SS	24H			48H			72H		
0	0.694	0.713	0.676	1.704	1.735	1.645	2.027	2.073	1.826
5	0.682	0.682	0.661	1.581	1.642	1.545	1.865	1.881	1.777
10	0.638	0.585	0.592	1.546	1.495	1.500	1.768	1.773	1.690
20	0.569	0.520	0.544	1.362	1.354	1.386	1.717	1.726	1.627
30	0.461	0.443	0.435	1.209	1.087	1.122	1.531	1.489	1.462
40	0.332	0.346	0.351	0.637	0.577	0.633	0.717	0.646	0.633
50	0.211	0.225	0.223	0.231	0.232	0.254	0.167	0.143	0.166

QGY-7703 — OD value(570 nM)

SS	24H			48H			72H		
0	0.942	0.930	0.882	1.232	1.234	1.016	1.459	1.415	1.397
5	0.921	0.868	0.879	1.145	1.179	1.099	1.596	1.585	1.381
10	0.858	0.913	0.805	1.181	1.197	1.160	1.688	1.549	1.610
20	0.737	0.755	0.662	0.887	0.909	0.928	1.378	1.436	1.357
30	0.427	0.406	0.422	0.690	0.585	0.743	1.049	1.216	1.088
40	0.245	0.246	0.234	0.316	0.341	0.345	0.295	0.399	0.271
50	0.112	0.114	0.120	0.143	0.127	0.121	0.129	0.118	0.126

Fig 2A

HepG2	One		Two		Three		Four	
	Con	SS	Con	SS	Con	SS	Con	SS
RQ value	1	1.598	1	2.538	1	2.76	1	1.59
CT value								
miR-375-3p	31.544	31.909	32.164	32.339	31.558	31.272	31.500	31.803
	31.317	31.378	36.114	31.260	31.702	31.513	34.545	31.409
	31.696	31.569	32.223	34.172	31.486	31.311	31.821	31.714
u6	13.067	13.830	12.789	13.404	12.904	13.021	12.878	13.096
	13.285	14.496	12.831	13.301	12.517	13.129	12.934	13.428
	13.132	13.485	12.727	12.945	12.715	13.137	12.680	13.124

Fig 2 A

QGY-7703	One			Two			Three	
	Con	SS		Con	SS		Con	SS
RQ value	1	3.099		1	3.075		1	2.64
CT value								
miR-375-3p	30.674	29.848		31.207	29.781		30.370	30.930
	30.154	29.893		31.668	29.577		30.573	30.961
	30.039	30.203		31.042	29.547		33.523	30.956
u6	12.374	13.521		13.080	12.469		12.659	13.454
	12.084	13.451		12.513	12.758		12.462	13.421
	12.264	13.623		12.540	12.754		12.444	13.271

Fig 2B

HepG2	One		Two		Three	
RQ value	CON	SS	CON	SS	CON	SS
	1	0.4249	1	0.6547	1	0.6874
CT value						
CCAT1	15.540	16.069	15.668	16.114	15.501	16.073
	15.136	15.520	15.099	15.630	15.237	15.678
	15.493	15.828	15.485	15.962	15.488	16.033
GAPDH	15.444	14.600	15.474	14.758	15.502	14.904
	15.153	14.991	15.206	15.073	15.274	15.093
	15.062	15.092	15.187	15.046	15.278	15.125

QGY-7703	One		Two		Three	
RQ value	CON	SS	CON	SS	CON	SS
	1	0.15918	1	0.56359	1	0.22313
CT value						
CCAT1	17.052	17.918	15.525	20.128	15.550	18.652
	17.097	17.920	15.646	20.166	15.746	18.779
	16.953	17.813	15.647	20.023	15.847	18.755
GAPDH	21.664	21.935	27.371	31.308	25.902	27.308
	29.643	24.784	24.349	28.491	23.251	24.810
	25.796	24.980	25.124	28.062	25.018	24.604

Fig 2 C	OD value(570 nM)						
	HepG2				QGY-7703		
Con	1.050	1.151	0.939		0.547	0.518	0.554
MO2	1.107	1.113	0.881		0.598	0.501	0.615
MO2-CCAT1	1.218	1.267	1.186		0.713	0.603	0.676

Fig 2 D	OD value(570 nM)						
	HepG2				QGY-7703		
Con	0.675	0.68	0.678		0.723	0.739	0.704
NC	0.612	0.643	0.611		0.693	0.749	0.71
miR-375-3p inhibitors	0.714	0.731	0.782		0.814	0.803	0.795

Fig 2 E											
HepG2		One				Two				Three	
	Con	NC	mimics		Con	NC	mimics		Con	NC	mimics
RQ value	1	0.539	0.354		1	0.904	0.239		1	0.892	0.433
CT value											
CCAT1	14.540	13.748	14.630		15.041	15.973	36.119		14.182	14.937	14.605
	14.574	13.592	14.692		14.977	15.999	36.119		13.469	13.943	14.476
	14.506	13.905	14.567						13.943	13.910	16.715
GAPDH	14.993	13.310	13.584		13.469	14.246	32.615		13.659	14.534	14.304
	16.579	13.125	13.399		13.739	14.625	32.685		14.220	14.344	14.521
	13.407	13.494	13.769						14.484	14.185	14.113

QGY-7703		One				Two				Three	
	Con	NC	mimics		Con	NC	mimics		Con	NC	mimics
RQ value	1	0.946	0.734		1	0.767	0.552		1	0.920	0.373
CT value											
CCAT1	20.804	20.828	21.187		21.868	21.771	22.345		21.142	21.167	22.878
	20.946	20.747	21.158		21.708	21.620	22.232		21.561	21.135	22.698
GAPDH	14.058	13.743	13.786		14.315	13.785	13.945		14.120	13.822	14.269
	13.945	13.925	13.921		14.332	13.914	13.992		14.285	13.942	14.164

Fig 2 F

HepG2		One				Two				Three	
	Con	MO2	CCAT1		Con	MO2	CCAT1		Con	MO2	CCAT1
RQ value	1	0.865	0.614		1	1.031	0.86		1	0.8139	0.6786
CT value											
miR-375-3p	32.207	31.054	31.603		31.952	32.056	32.379		31.250	31.444	31.544
	32.094	31.336	31.673		31.826	31.585	31.855		31.091	31.224	31.544
u6	12.188	11.077	10.982		10.698	10.654	10.638		9.329	9.188	9.164
	12.394	11.174	11.169		10.838	10.833	10.921		9.385	9.259	9.176

QGY-7703		One				Two				Three				Four	
	Con	MO2	CCAT1		Con	MO2	CCAT1		Con	MO2	CCAT1		Con	MO2	CCAT1
RQ value	1	1.051462	0.833404		1	1.19806	0.67014		1	0.798	0.563		1	0.921	0.4
CT value															
miR-375-3p	22.413	21.892	22.258		21.757	21.975	22.979		21.950	22.438	25.158		21.941	21.703	22.916
	22.497	22.051	22.536		21.929	22.120	23.282		22.023	22.486	23.059		22.073	21.931	23.058
u6	10.206	9.875	9.955		9.579	10.194	10.374		10.473	10.651	12.963		8.310	7.957	7.995
	10.426	9.935	10.035		10.013	10.329	10.638		10.588	10.710	10.686		8.354	8.091	7.996

Figure 3A

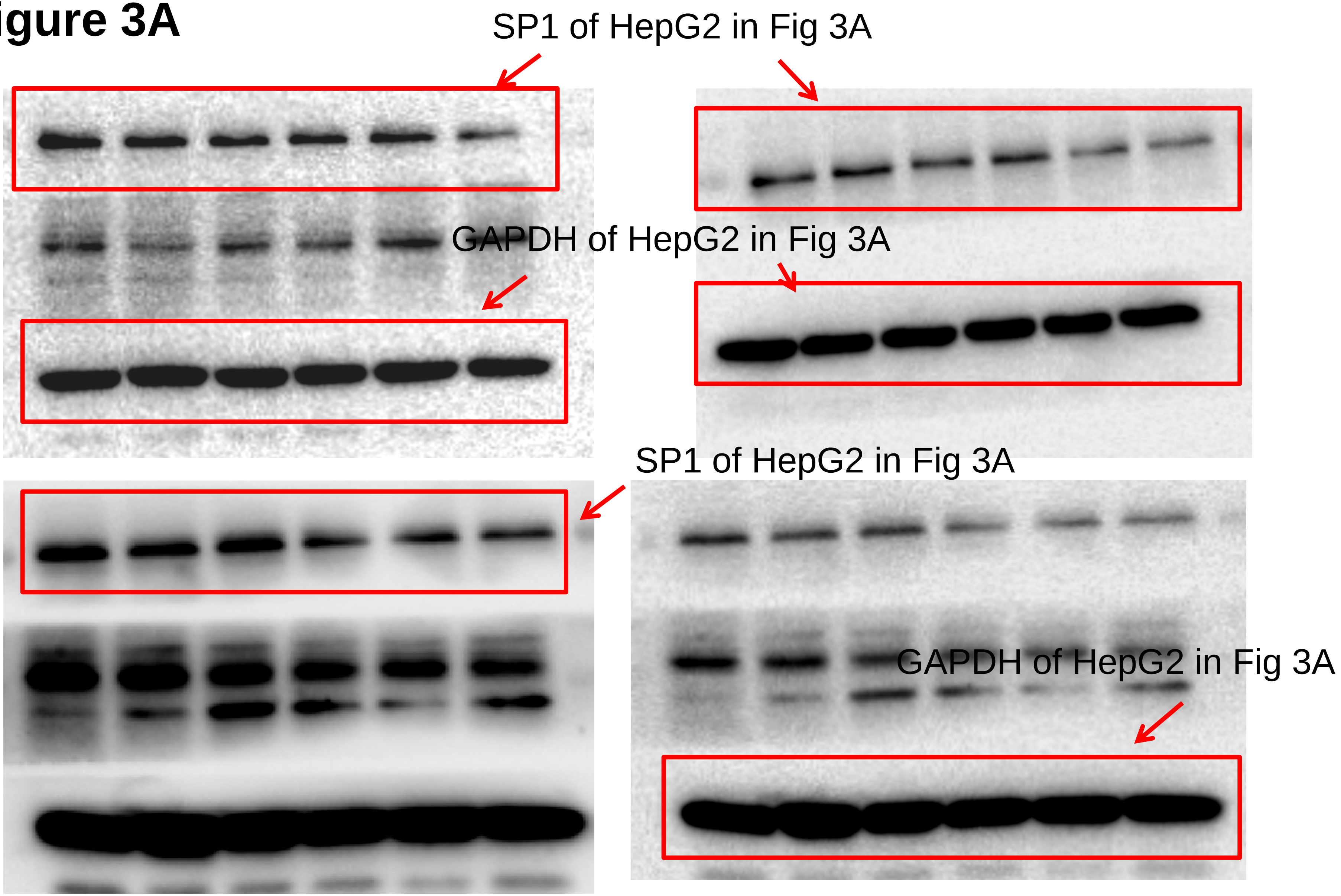
SP1 of HepG2 in Fig 3A
GAPDH of HepG2 in Fig 3A
SP1 of HepG2 in Fig 3A
GAPDH of HepG2 in Fig 3A

Figure 3A

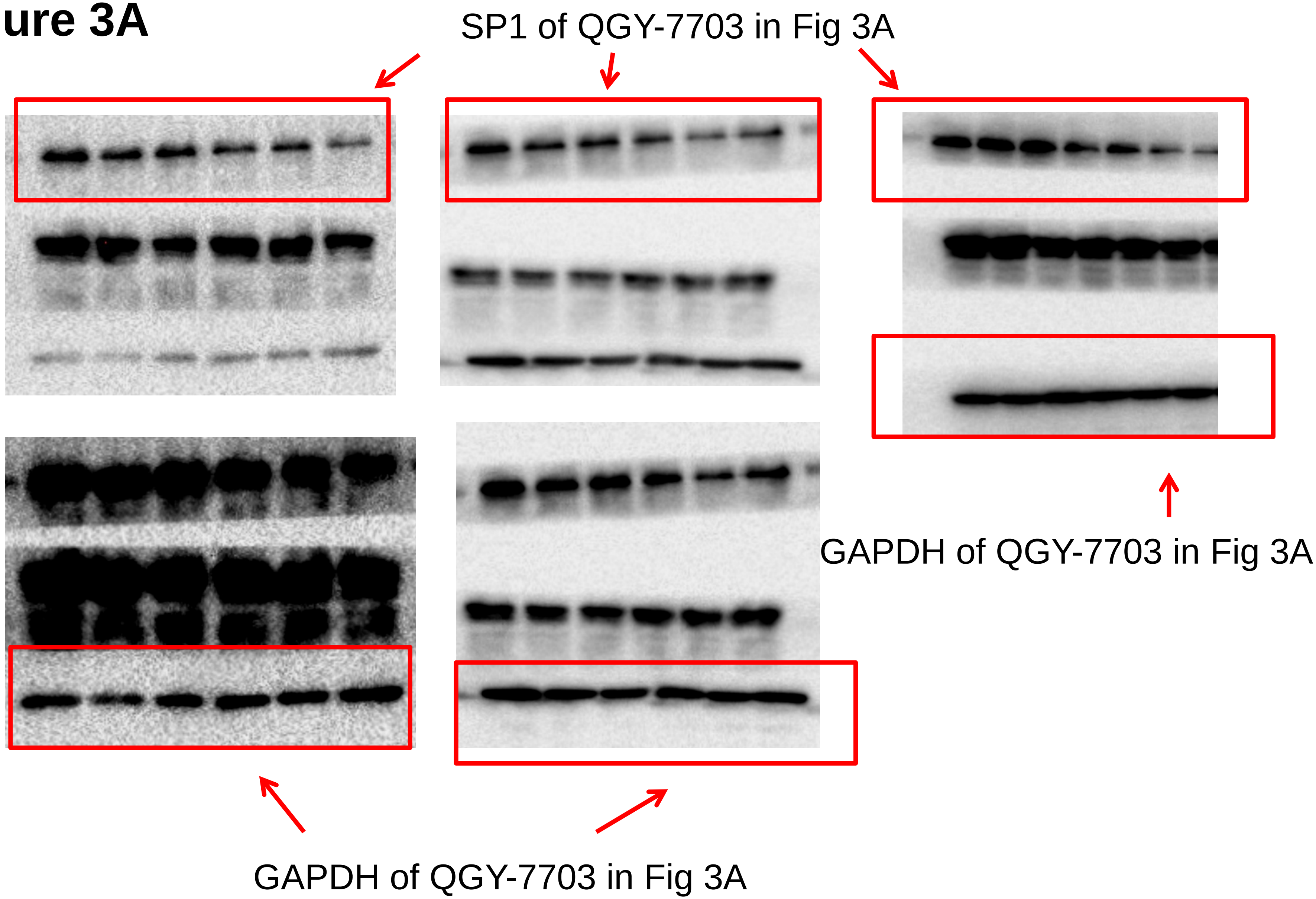
SP1 of QGY-7703 in Fig 3A
GAPDH of QGY-7703 in Fig 3A
GAPDH of QGY-7703 in Fig 3A

Fig 3B

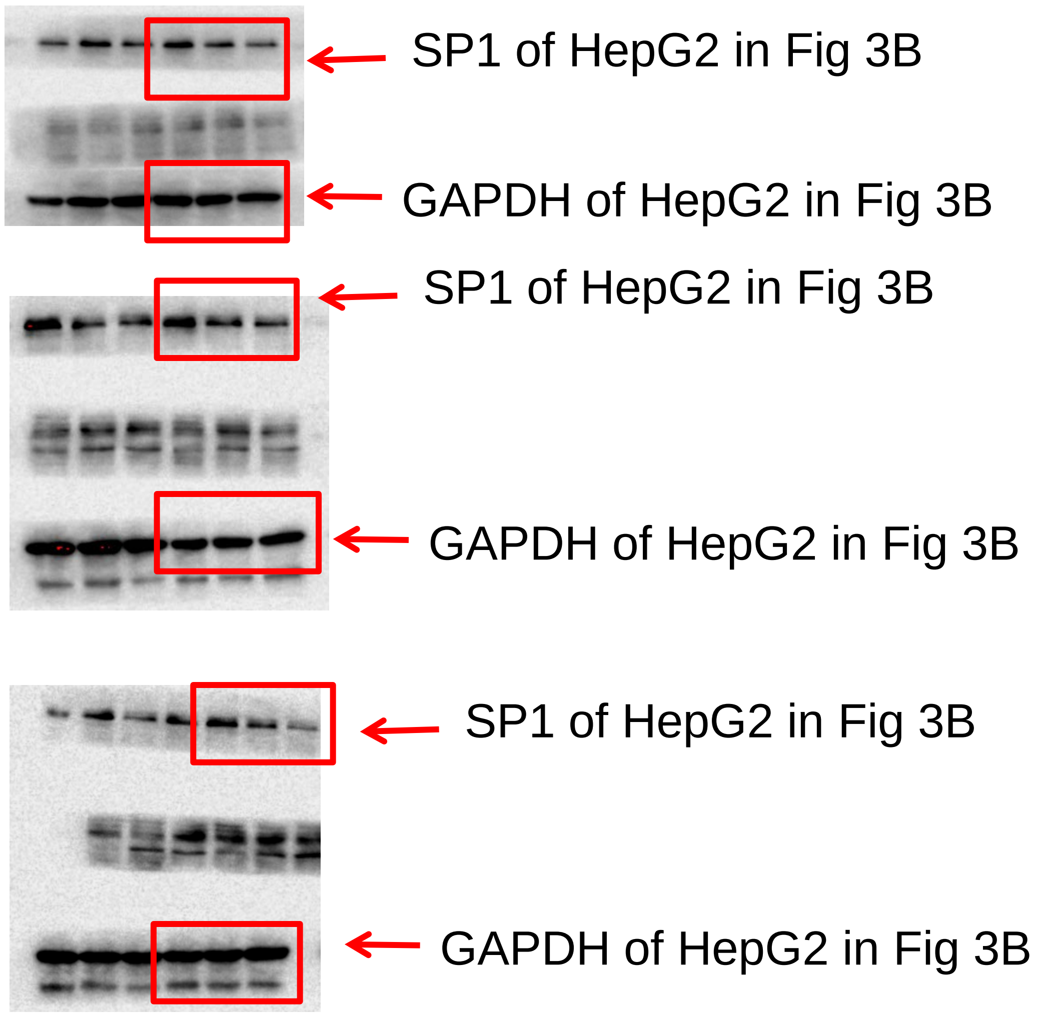
SP1 of HepG2 in Fig 3B
GAPDH of HepG2 in Fig 3B
SP1 of HepG2 in Fig 3B
GAPDH of HepG2 in Fig 3B
SP1 of HepG2 in Fig 3B
GAPDH of HepG2 in Fig 3B

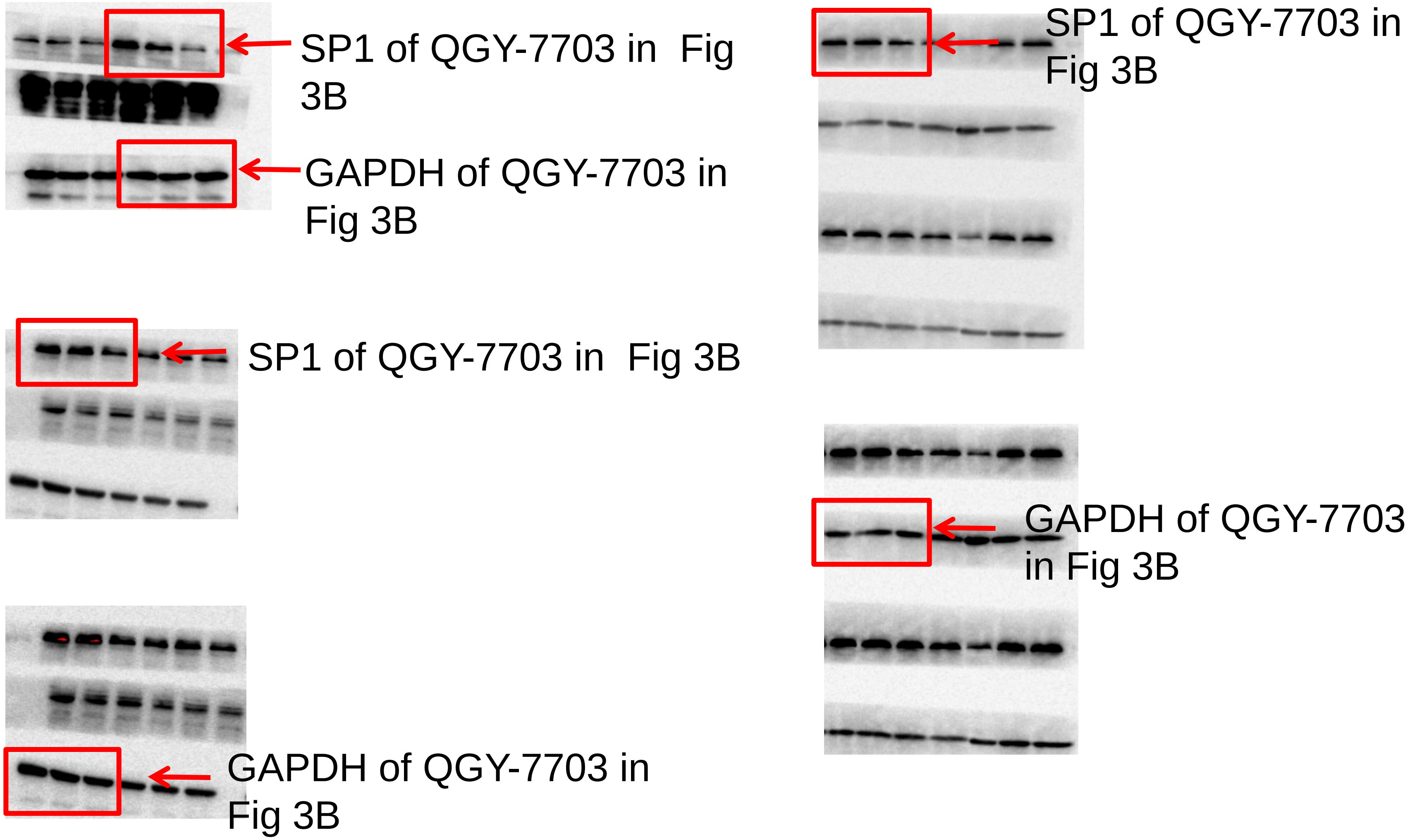
SP1 of QGY-7703 in Fig 3B
GAPDH of QGY-7703 in Fig 3B
SP1 of QGY-7703 in Fig 3B
GAPDH of QGY-7703 in Fig 3B
SP1 of QGY-7703 in Fig 3B
GAPDH of QGY-7703 in Fig 3B

Fig 3C

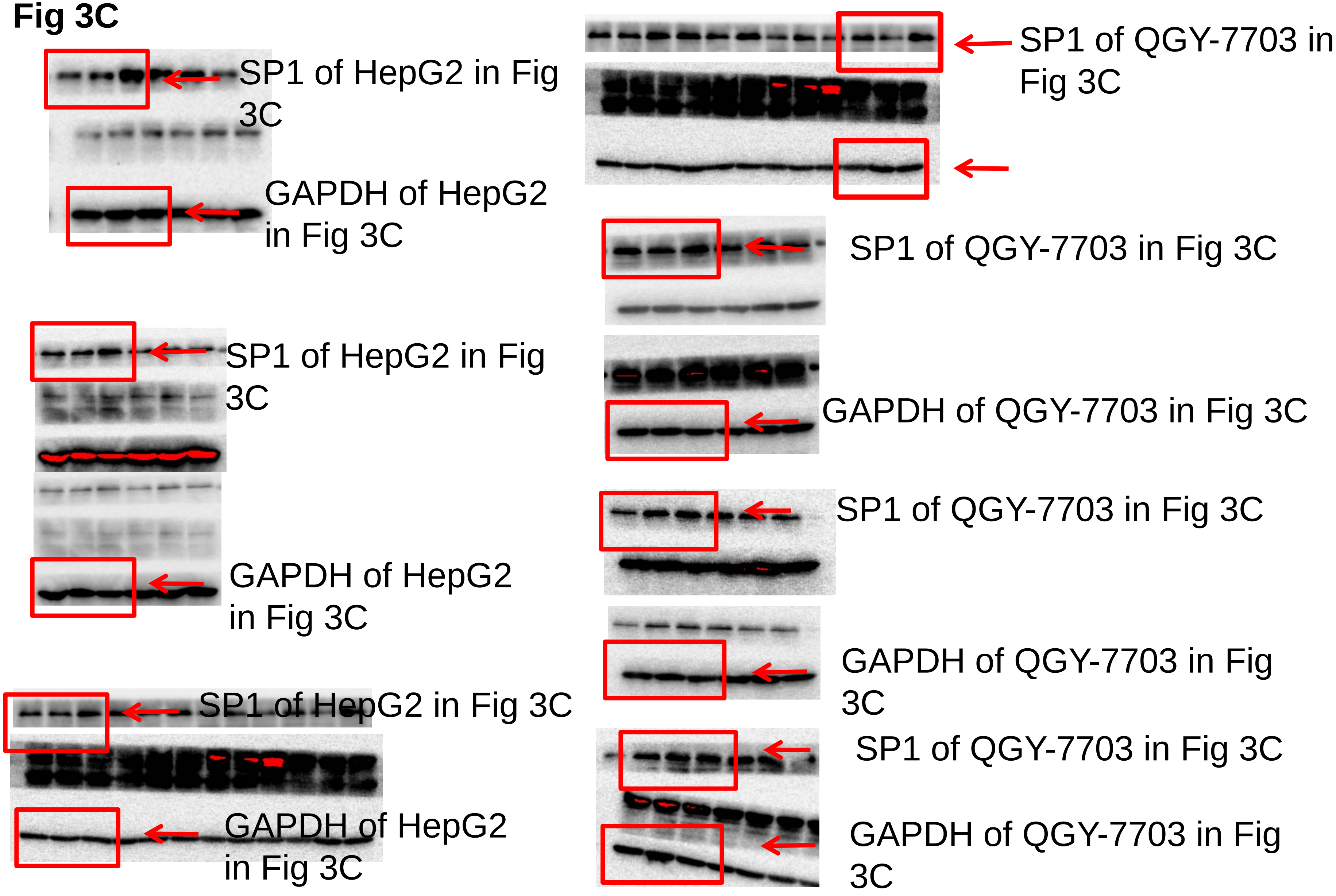

SP1 of HepG2 in Fig 3C
GAPDH of HepG2 in Fig 3C
SP1 of HepG2 in Fig 3C
GAPDH of HepG2 in Fig 3C
SP1 of HepG2 in Fig 3C
GAPDH of HepG2 in Fig 3C
SP1 of QGY-7703 in Fig 3C
SP1 of QGY-7703 in Fig 3C
GAPDH of QGY-7703 in Fig 3C
SP1 of QGY-7703 in Fig 3C
GAPDH of QGY-7703 in Fig 3C
SP1 of QGY-7703 in Fig 3C
GAPDH of QGY-7703 in Fig 3C

Fig 3D

HepG2	One					
	Con	pcDNA3.1	pcDNA3.1-SP1	SS	pcDNA3.1+SS	pcDNA3.1-SP1+SS
RQ value	1	0.890	0.830	0.695	0.631	0.702
CT value						
CCAT1	14.869	14.946	15.124	16.375	15.662	15.437
	14.784	15.053	15.120	16.401	15.681	15.506
GAPDH	13.908	13.940	13.910	14.971	14.145	14.055
	14.022	14.000	14.074	15.032	14.144	14.142

	Two					
	Con	pcDNA3.1	pcDNA3.1-SP1	SS	pcDNA3.1+SS	pcDNA3.1-SP1+SS
RQ value	1	0.892	0.867	0.887	0.715	1.025
CT value						
CCAT1	15.550	15.561	15.594	15.478	15.351	15.496
	15.520	15.570	15.553	15.230	15.308	15.565
GAPDH	13.926	13.837	13.743	13.603	13.257	14.037
	13.983	13.803	13.833	13.598	13.272	13.934

	Three					
	Con	pcDNA3.1	pcDNA3.1-SP1	SS	pcDNA3.1+SS	pcDNA3.1-SP1+SS
RQ value	1	0.924	1.4	0.86	0.776	0.996
CT value						
CCAT1	14.507	14.853	14.495	15.162	15.691	15.490
	14.516	14.920	14.579	15.185	15.661	15.447
GAPDH	13.526	13.673	13.857	13.802	14.092	14.326
	13.058	13.643	13.834	13.668	14.089	14.161

	Four					
	Con	pcDNA3.1	pcDNA3.1-SP1	SS	pcDNA3.1+SS	pcDNA3.1-SP1+SS
RQ value	1.000	1.154	0.940	0.963	0.906	1.083
CT value						
CCAT1	15.718	15.330	15.833	15.649	15.048	15.496
	15.659	15.400	15.781	15.567	14.990	15.565
GAPDH	13.960	13.855	14.014	13.853	13.182	14.037
	14.004	13.875	14.007	13.840	13.159	13.842

	Five					
	Con	pcDNA3.1	pcDNA3.1-SP1	SS	pcDNA3.1+SS	pcDNA3.1-SP1+SS
RQ value	1	0.57	0.61	0.82	0.49	1.12
CT value						
CCAT1	17.194	15.762	16.000	17.763	16.677	16.779
	17.195	15.789	16.021	17.782	16.670	16.639
GAPDH	16.477	14.263	14.537	16.765	14.933	16.233
	16.443	14.205	14.588	16.748	14.899	16.056

QGY-7703	One					
	Con	pcDNA3.1	pcDNA3.1-SP1	SS	pcDNA3.1+SS	pcDNA3.1-SP1+SS
RQ value	1	0.618	0.623	0.524	0.479	0.607
CT value						
CCAT1	21.630	21.834	21.812	24.643	23.002	23.428
	21.528	21.761	21.850	24.648	22.977	23.349
GAPDH	14.417	13.971	14.022	16.579	14.804	15.438
	14.466	13.959	14.059	16.559	14.777	15.624

	Two					
	Con	pcDNA3.1	pcDNA3.1-SP1	SS	pcDNA3.1+SS	pcDNA3.1-SP1+SS
RQ value	1	0.86	0.66	0.33	0.333	0.45
CT value						
CCAT1	21.923	21.751	22.404	31.192	30.603	31.829
	21.977	21.848	22.409	31.347	30.396	32.314
GAPDH	14.527	14.103	14.356	22.220	21.319	23.421
	14.464	14.154	14.346	22.218	21.595	23.506

	Three					
	Con	pcDNA3.1	pcDNA3.1-SP1	SS	pcDNA3.1+SS	pcDNA3.1-SP1+SS
RQ value	1	0.816	0.88	0.497	0.459	0.641
CT value						
CCAT1	22.345	22.181	21.988	23.732	22.439	22.591
	22.394	22.315	21.999	23.765	22.593	22.622
GAPDH	14.686	14.208	13.977	15.004	13.666	14.133
	14.607	14.254	14.197	15.032	13.676	14.352

Fig 3E

HepG2	One					
	Con	pcDNA3.1	pcDNA3.1-SP1	SS	pcDNA3.1+SS	pcDNA3.1-SP1+SS
RQ value	1	0.986	0.795	1.675	1.201	0.842
CT value						
miR-375-3p	31.947	31.391	32.771	31.286	32.142	32.977
	31.662	31.231	32.611	31.156	31.709	31.527
u6	8.086	7.764	8.510	8.174	8.446	8.167
	7.943	7.769	8.631	8.177	8.353	8.263

	Two					
	Con	pcDNA3.1	pcDNA3.1-SP1	SS	pcDNA3.1+SS	pcDNA3.1-SP1+SS
RQ value	1	1.021	1.097	1.734	1.486	0.64
CT value						
miR-375-3p	31.606	31.775	32.010	31.324	31.613	31.119
	31.929	32.192	31.424	31.001	31.570	33.883
u6	8.253	8.472	8.282	8.449	8.533	8.453
	8.264	8.536	8.400	8.447	8.774	8.241

	Three					
	Con	pcDNA3.1	pcDNA3.1-SP1	SS	pcDNA3.1+SS	pcDNA3.1-SP1+SS
RQ value	1.000	0.892	0.993	1.890	1.201	0.963
CT value						
miR-375-3p	31.722	31.975	31.669	32.440	32.242	32.156
	32.061	31.564	31.609	32.515	31.985	31.377
u6	8.841	8.552	8.512	10.324	9.382	8.679
	8.874	8.589	8.677	10.400	9.305	8.676

QGY-7703				One		
	Con	pcDNA3.1	pcDNA3.1-SP1	SS	pcDNA3.1+SS	pcDNA3.1-SP1+SS
RQ value	1.000	1.048	0.552	2.034	1.640	1.101
CT value						
miR-375-3p	24.963	24.674	24.940	25.599	24.662	24.622
	24.650	24.889	24.634	25.066	24.673	24.600
u6	9.692	9.805	8.691	11.335	10.336	9.579
	9.645	9.617	8.894	11.103	10.150	9.643

				Two		
	Con	pcDNA3.1	pcDNA3.1-SP1	SS	pcDNA3.1+SS	pcDNA3.1-SP1+SS
RQ value	1.000	0.905	1.110	2.141	1.359	1.096
CT value						
miR-375-3p	24.618	24.384	24.082	24.742	24.162	24.392
	24.570	24.434	23.976	24.695	24.078	24.482
u6	7.765	7.379	7.340	8.943	7.735	7.672
	7.713	7.441	7.309	8.980	7.680	7.758

				Three		
	Con	pcDNA3.1	pcDNA3.1-SP1	SS	pcDNA3.1+SS	pcDNA3.1-SP1+SS
RQ value	1.000	1.010	1.054	2.006	1.361	0.954
CT value						
miR-375-3p	24.324	24.527	24.128	24.405	24.224	24.630
	24.063	24.173	23.908	24.335	24.233	24.471
u6	7.248	7.514	7.249	8.442	7.646	7.509
	7.319	7.396	7.120	8.486	7.879	7.636

Fig 4 A

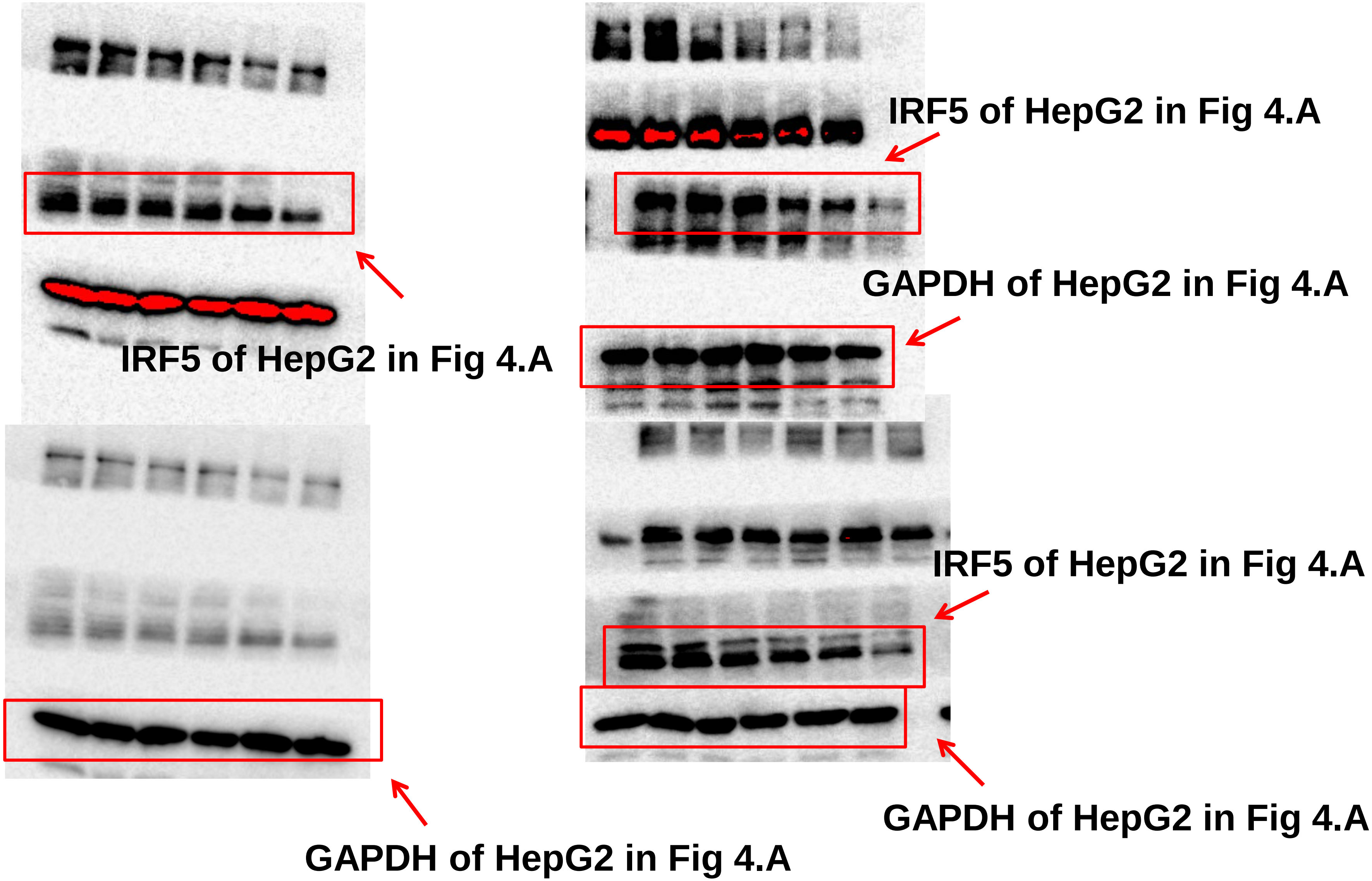
IRF5 of HepG2 in Fig 4.A
GAPDH of HepG2 in Fig 4.A
IRF5 of HepG2 in Fig 4.A
GAPDH of HepG2 in Fig 4.A
IRF5 of HepG2 in Fig 4.A
GAPDH of HepG2 in Fig 4.A

Fig 4 A

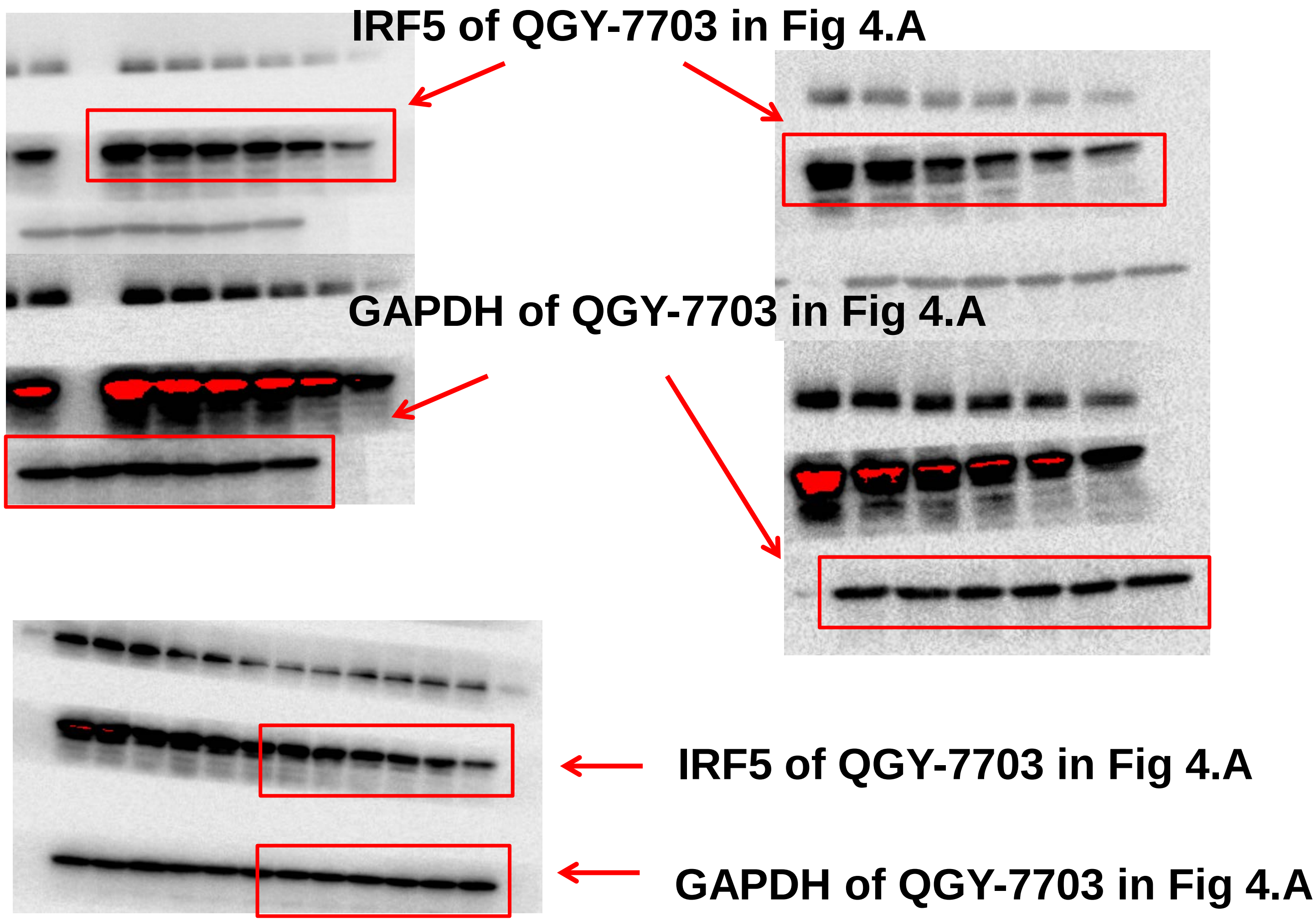
IRF5 of QGY-7703 in Fig 4.A
GAPDH of QGY-7703 in Fig 4.A
IRF5 of QGY-7703 in Fig 4.A
GAPDH of QGY-7703 in Fig 4.A

Fig 4B 4C

	HepG2				
	**Gluc value**	**SEAP value**		**Gluc/SEAP**	**Relative to Con**
**IRF5 promoter**	14052	963891		0.014578412	1
	18637	1194702		0.015599706	1
	13092	834116		0.015695659	1
**IRF5 promoter+SS**	11879	1038442		0.011439252	0.781978174
	7542	568436		0.013267984	0.850527852
	7176	524424		0.013683584	0.871806929

	HepG2				
	**Gluc value**	**SEAP value**		**Gluc/SEAP**	**Relative to Con**
**IRF5 promoter**	14052	963891		0.014578412	1
	18637	1194702		0.015599706	1
	13092	834116		0.015695659	1
**IRF5 promoter+pcDNA3.1**	19063	1361287		0.01400366	1.030617607
	20386	1277777		0.015954271	0.960575114
	20617	1274525		0.016176223	1.022728927
**IRF5 promoter+pcDNA3.1-SP1**	19048	1347477		0.014136048	1.017543944
	21804	1394208		0.015638986	0.969656252
	22571	1413247		0.015971023	1.002518024
**IRF5 promoter+SS**	11879	1038442		0.011439252	0.781978174
	7542	568436		0.013267984	0.850527852
	7176	524424		0.013683584	0.871806929
**IRF5 promoter+pcDNA3.1+SS**	8363	711325		0.011756932	0.806461807
	7142	786117		0.009085162	0.582393131
	14180	1170774		0.012111646	0.770913792
**IRF5 promoter+pcDNA3.1-SP1+[illegible]**	10378	698073		0.01486664	0.902253785
	9094	646115		0.014074894	1.019770879
	8867	593105		0.014950135	0.952501281

Application: Tecan i-control | Tecan i-control , 1.10.4.0
Device: infinite M1000Pro | Serial number: 1211011466
Firmware: V_1.05_11/2011_S3LCE_ALPHA (Nov 3 2011/09.27.24) | MAI, V_1.05_11/2011_S3LCE_ALPHA (Nov 3 2011/09.27.24)

Date: 2019-3-3
Time: 21:37:45

System: TECAN
User: TECAN\Administrator
Plate: Costar 96 Flat Bottom White Polystyrol Catalog No.: 3600/3362/3917/3912/3922/3596 [COS96fw.pdfx]
Plate-ID (Stacker)

Shaking (Linear) Duration: 10 s
Shaking (Linear) Amplitude: 2 mm
Shaking (Linear) Frequency: 654 rpm

Label: Label1
Mode: Luminescence
Attenuation: NONE
Integration Time: 1000 ms
Settle Time: 0 ms
Part of Plate: A7-H12
Start Time: 2019/3/3 21:38:04

Temperature: 25.8 ° C

<>	7	8	9	10	11	12
A	14052	18637	13092	11879	7542	7176
B	19063	20386	20617	8363	7142	14180
C	19048	21804	22571	10378	9094	8867
D						
E	963891	1194702	834116	1038442	568436	524424
F	1361287	1277777	1274525	711325	786117	1170774
G	1347477	1394208	1413247	698073	646115	593105
H						

	QGY-7703				
	**Gluc value**	**SEAP value**		**Gluc/SEAP**	**Relative to Con**
**IRF5 promoter**	6429	609960		0.010540035	1
	5839	514303		0.01135323	1
	5157	434652		0.011864664	1
**IRF5 promoter+SS**	7690	804833		0.009554777	0.906522306
	8681	860345		0.010090138	0.888746083
	8905	863722		0.01031003	0.868969419

Application: Tecan i-control			Tecan i-control , 1.10.4.0			
Device: infinite M1000Pro			Serial number: 1211011466			
Firmware: V_1.05_11/2011_S3LCE_ALPHA (Nov 3 2011/09.27.24)			MAI, V_1.05_11/2011_S3LCE_ALPHA (Nov 3 2011/09.27.24)			
Date:	2019-1-16					
Time:	11:40:47					
System			TECAN			
User			TECAN\Administrator			
Plate			Costar 96 Flat Bottom White Polystyrol Catalog No.: 3600/3362/3917/3912/3922/3596 [COS96fw.pdfx]			
Plate-ID (Stacker)						
Shaking (Linear) Duration:					15	s
Shaking (Linear) Amplitude:					2	mm
Shaking (Linear) Frequency:					654	rpm
Label: Label1						
Mode				Luminescence		
Attenuation				NONE		
Integration Time					1000	ms
Settle Time					0	ms
Part of Plate				E7-H12		
Start Time:	2019/1/16 11:41:11					
	Temperature: 22.9 °C					

<>	7	8	9	10	11	12
E	6429	5839	5157	7690	8681	8905
F						
G	609960	514303	434652	804833	860345	863722
H						

	QGY-7703				
	**Gluc value**	**SEAP value**		**Gluc/SEAP**	**Relative to Con**
**IRF5 promoter**	7981	312701		0.025522784	1
	7427	285540		0.026010366	1
	6724	257580		0.026104511	1
**IRF5 promoter+pcDNA3.1**	15165	586472		0.025858012	1.01313447
	9745	372089		0.026189971	1.006905107
	10035	351091		0.028582333	1.094919308
**IRF5 promoter+pcDNA3.1-SP1**	13295	465721		0.028547134	1.118496114
	12495	463981		0.026929982	1.035355739
	12736	528913		0.024079575	0.922429626
**IRF5 promoter+SS**	7294	445929		0.016356864	0.640873039
	13118	615504		0.021312615	0.819389281
	10028	518806		0.019328998	0.740446644
**IRF5 promoter+pcDNA3.1+SS**	11673	528797		0.022074634	0.864899134
	10027	485550		0.020650808	0.79394531
	9089	407044		0.022329281	0.855380174
**IRF5 promoter+pcDNA3.1-SP1+SS**	14677	585557		0.025065024	0.982064645
	14722	585497		0.02514445	0.966708797
	11179	415243		0.026921586	1.031300125

Application: Tecan i-control			Tecan i-control , 1.10.4.0			
Device: infinite M1000Pro			Serial number: 1211011466			
Firmware: V_1.05_11/2011_S3LCE_ALPHA (Nov 3 2011/09.27.24)			MAI, V_1.05_11/2011_S3LCE_ALPHA (Nov 3 2011/09.27.24)			
Date:	2019-3-15					
Time:	10:21:40					
System			TECAN			
User			TECAN\Administrator			
Plate			Costar 96 Flat Bottom White Polystyrol Catalog No.: 3600/3362/3917/3912/3922/3596 [COS96fw.pdfx]			
Plate-ID (Stacker)						
Shaking (Linear) Duration:				15	s	
Shaking (Linear) Amplitude:				2	mm	
Shaking (Linear) Frequency:				654	rpm	
Label: Label1						
Mode				Luminescence		
Attenuation				NONE		
Integration Time				1000	ms	
Settle Time				0	ms	
Part of Plate				A1-H6		
Start Time:	2019/3/15 10:22:04					
	Temperature: 22 °C					

<>	1	2	3	4	5	6
A	7981	7427	6724	7294	13118	10028
B	15165	9745	10035	11673	10027	9089
C	13295	12495	12736	14677	14722	11179
D						
E						
F	312701	285540	257580	445929	615504	518806
G	586472	372089	351091	528797	485550	407044
H	465721	463981	528913	585557	585497	415243

Fig 4D HepG2

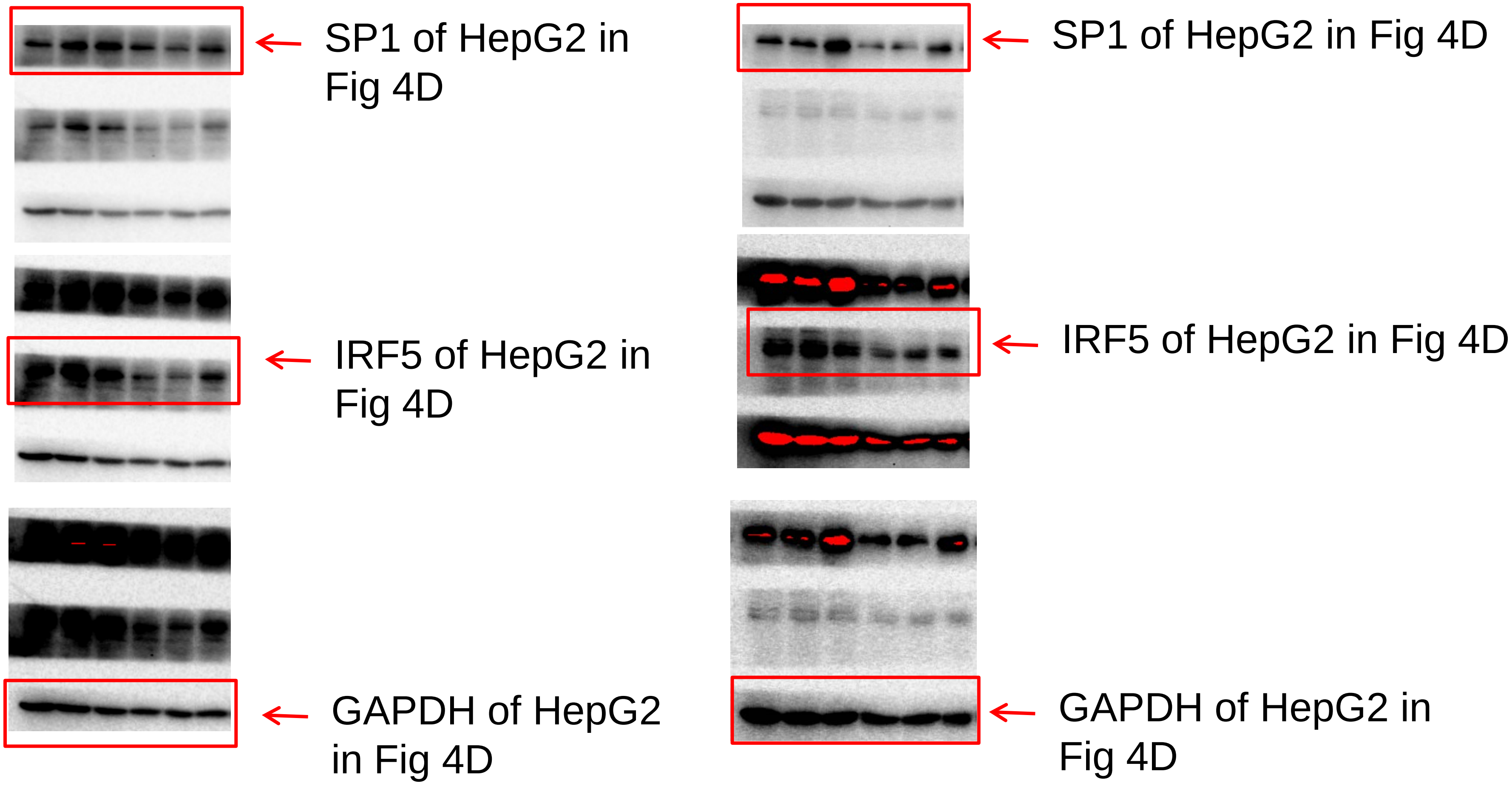
SP1 of HepG2 in Fig 4D
IRF5 of HepG2 in Fig 4D
GAPDH of HepG2 in Fig 4D
SP1 of HepG2 in Fig 4D
IRF5 of HepG2 in Fig 4D
GAPDH of HepG2 in Fig 4D

HepG2

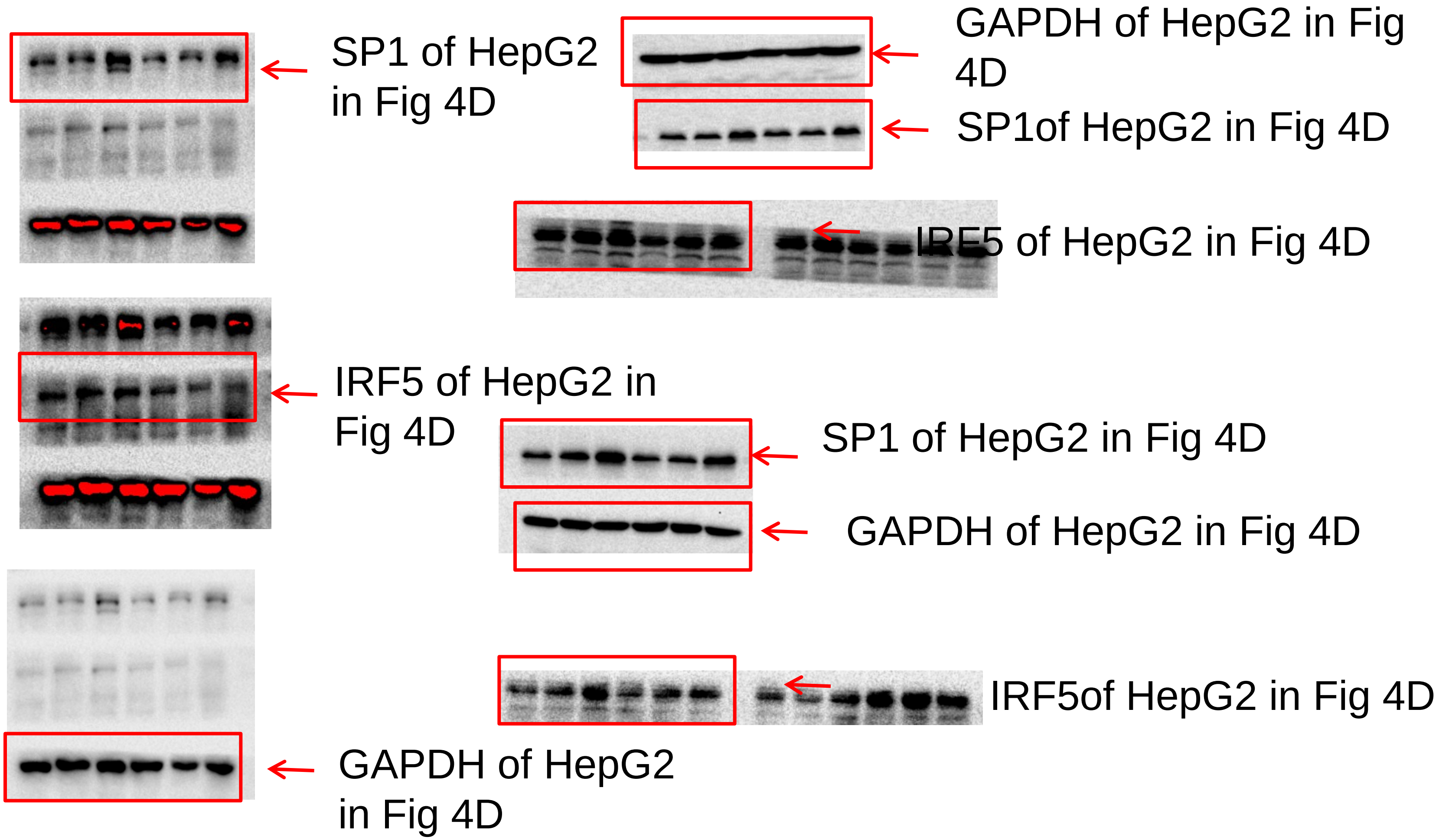
SP1 of HepG2 in Fig 4D
GAPDH of HepG2 in Fig 4D
SP1of HepG2 in Fig 4D
IRF5 of HepG2 in Fig 4D
IRF5 of HepG2 in Fig 4D
SP1 of HepG2 in Fig 4D
GAPDH of HepG2 in Fig 4D
IRF5of HepG2 in Fig 4D
GAPDH of HepG2 in Fig 4D

QGY-7703

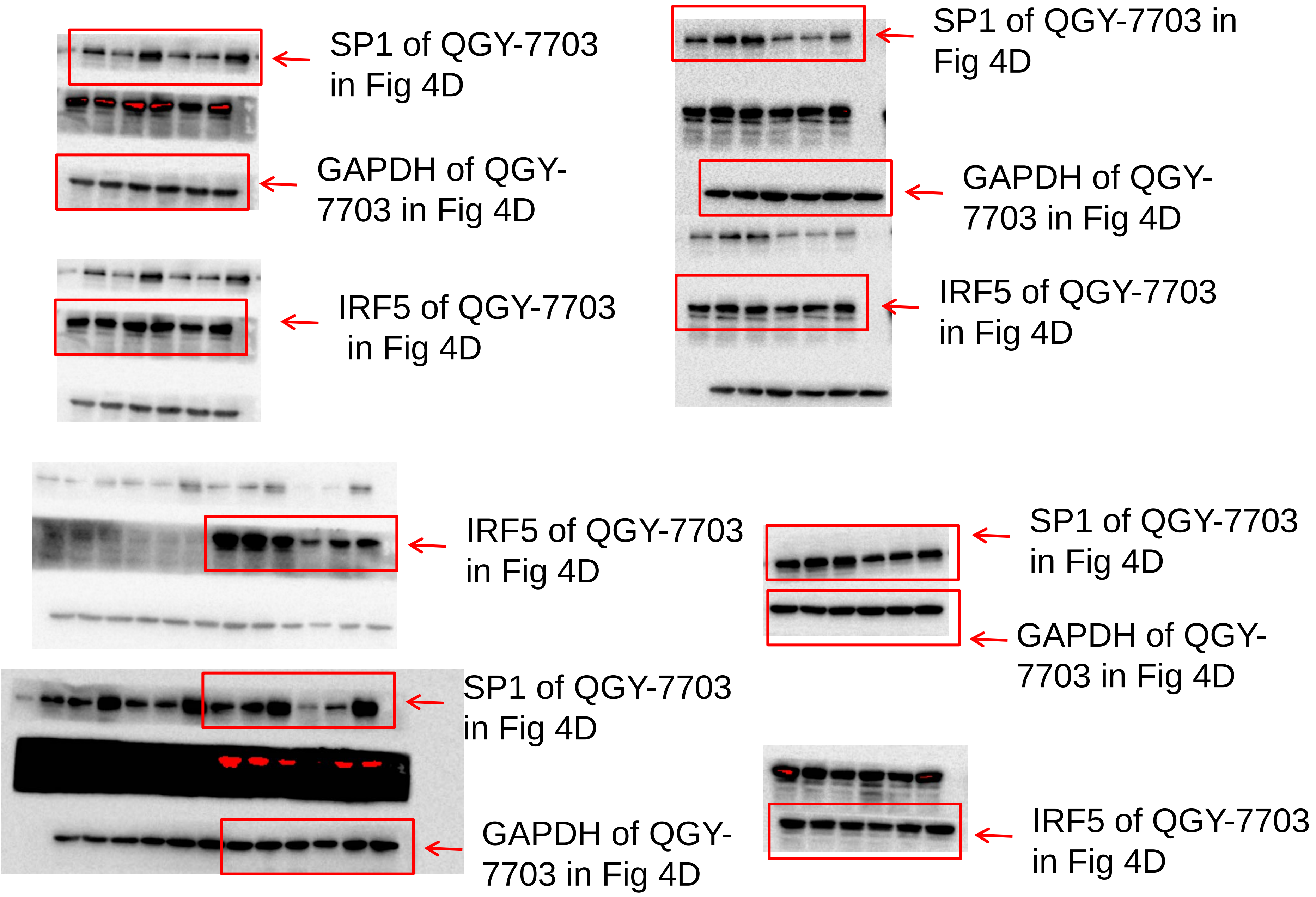

SP1 of QGY-7703 in Fig 4D
GAPDH of QGY-7703 in Fig 4D
IRF5 of QGY-7703 in Fig 4D
SP1 of QGY-7703 in Fig 4D
GAPDH of QGY-7703 in Fig 4D
IRF5 of QGY-7703 in Fig 4D
IRF5 of QGY-7703 in Fig 4D
SP1 of QGY-7703 in Fig 4D
GAPDH of QGY-7703 in Fig 4D
SP1 of QGY-7703 in Fig 4D
GAPDH of QGY-7703 in Fig 4D
IRF5 of QGY-7703 in Fig 4D

Fig 5 A

HepG2	One					
	Con	pEZ-MO2	pEZ-MO2-IRF5	SS	pEZ-MO2+SS	pEZ-MO2-IRF5+SS
RQ value	1	0.909	0.992	0.755	0.806	0.956
CT value						
CCAT1	14.526	14.426	14.257	14.771	14.711	14.586
	14.376	14.382	14.300	14.600	14.505	14.267
GAPDH	13.225	12.936	12.876	12.975	12.980	12.989
	13.070	12.991	13.052	12.978	13.007	13.128

	Two					
	Con	pEZ-MO2	pEZ-MO2-IRF5	SS	pEZ-MO2+SS	pEZ-MO2-IRF5+SS
RQ value	1	1.022	0.983	0.736	0.624	1.024
CT value						
CCAT1	14.023	14.158	13.830	14.756	14.762	14.006
	13.881	13.629	14.281	14.367	14.586	13.936
GAPDH	12.708	12.724	12.829	12.734	12.883	12.928
	12.943	12.873	12.982	13.251	12.854	12.829

	Three					
	Con	pEZ-MO2	pEZ-MO2-IRF5	SS	pEZ-MO2+SS	pEZ-MO2-IRF5+SS
RQ value	1	0.967	1.002	0.677	0.888	0.962
CT value						
CCAT1	16.378	16.026	16.484	16.768	16.790	16.699
	16.229	15.852	16.183	16.626	16.767	16.861
GAPDH	15.340	14.853	15.258	15.074	15.600	15.551
	15.234	14.896	15.381	15.163	15.581	15.864

QGY-7703	One					
	Con	pEZ-MO2	pEZ-MO2-IRF5	SS	pEZ-MO2+SS	pEZ-MO2-IRF5+SS
RQ value	1	0.842	0.816	0.282	0.58	0.698
CT value						
CCAT1	21.340	21.084	21.417	22.827	21.808	21.533
	20.737	21.443	21.455	22.495	21.823	21.680
GAPDH	12.546	12.589	12.657	12.325	12.636	12.710
	12.636	12.548	12.734	12.451	12.528	12.573

	Two					
	Con	pEZ-MO2	pEZ-MO2-IRF5	SS	pEZ-MO2+SS	pEZ-MO2-IRF5+SS
RQ value	1	0.853	0.782	0.349	0.537	0.83
CT value						
CCAT1	20.622	20.574	20.556	22.412	21.771	21.001
	20.476	20.379	20.302	22.325	21.829	20.905
	20.289	20.255	20.171	22.531	21.719	20.956
GAPDH	12.976	12.711	12.372	13.931	13.650	13.421
	13.001	12.729	12.582	13.554	13.445	13.218
	13.224	12.895	12.825	13.039	13.345	13.230

	Three					
	Con	pEZ-MO2	pEZ-MO2-IRF5	SS	pEZ-MO2+SS	pEZ-MO2-IRF5+SS
RQ value	1	0.812	0.719	0.426	0.626	0.933
CT value						
CCAT1	20.621	21.362	20.389	21.829	20.958	20.882
	20.488	21.180	20.426	21.692	20.932	21.150
GAPDH	13.427	13.869	12.582	13.169	12.703	13.155
	12.908	13.299	12.509	13.116	13.063	13.901

Fig 5 B

HepG2	One					
	Con	pEZ-MO2	pEZ-MO2-IRF5	SS	pEZ-MO2+SS	pEZ-MO2-IRF5+SS
RQ value	1	0.709	0.821	1.25	1.387	1.147
CT value						
miR-375-3p	32.445	32.303	30.795	31.431	31.492	30.889
	31.748	31.422	30.898	31.413	30.773	30.478
	31.653	31.906	31.088	31.449	27.676	30.913
u6	12.369	11.742	10.938	12.092	10.932	11.242
	12.464	11.843	11.102	12.077	10.811	11.391
	12.424	11.966	11.299	12.501	11.026	11.652

	Two					
	Con	pEZ-MO2	pEZ-MO2-IRF5	SS	pEZ-MO2+SS	pEZ-MO2-IRF5+SS
RQ value	1	0.859	0.963	1.833	1.200	1.114
CT value						
miR-375-3p	31.389	31.853	31.579	32.279	31.779	31.811
	31.008	31.443	31.142	32.165	31.849	31.306
u6	9.074	9.283	9.166	10.962	9.945	9.561
	9.062	9.314	9.185	10.971	9.948	9.606

	Three					
	Con	pEZ-MO2	pEZ-MO2-IRF5	SS	pEZ-MO2+SS	pEZ-MO2-IRF5+SS
RQ value	1	0.921	0.898	1.916	1.397	0.956
CT value						
miR-375-3p	31.652	31.401	31.796	32.604	32.054	31.760
	32.058	31.791	31.542	32.383	32.116	31.680
u6	8.851	8.351	8.601	10.411	9.506	8.917
	8.926	8.667	8.494	10.518	9.695	8.471

	Four					
	Con	pEZ-MO2	pEZ-MO2-IRF5	SS	pEZ-MO2+SS	pEZ-MO2-IRF5+SS
RQ value	1	1.197	1.046	1.776	1.599	1.147
CT value						
miR-375-3p	31.396	31.404	31.813	31.715	31.514	32.319
	31.451	31.398	32.001	31.694	31.564	32.087
u6	7.652	7.820	8.153	8.757	8.429	8.559
	7.530	7.837	8.128	8.645	8.340	8.579

QGY-7703	One					
	Con	pEZ-MO2	pEZ-MO2-IRF5	SS	pEZ-MO2+SS	pEZ-MO2-IRF5+SS
RQ value	1	0.838	0.854	1.644	1.365	1.19461
CT value						
miR-375-3p	21.497	22.030	21.432	21.292	21.489	21.969
	21.315	21.924	21.344	21.209	21.517	21.933
u6	10.186	10.345	9.656	10.604	10.502	10.905
	10.094	10.570	10.134	10.800	10.869	10.978
	Two					
	Con	pEZ-MO2	pEZ-MO2-IRF5	SS	pEZ-MO2+SS	pEZ-MO2-IRF5+SS
RQ value	1	1.013	1.192	1.64	1.43	1.272
CT value						
miR-375-3p	21.784	21.741	21.344	21.339	21.705	21.868
	21.728	21.683	21.198	21.272	21.669	22.150
u6	7.793	7.804	7.515	7.974	8.289	8.845
	7.938	7.878	7.753	8.283	8.335	8.085

	Three					
	Con	pEZ-MO2	pEZ-MO2-IRF5	SS	pEZ-MO2+SS	pEZ-MO2-IRF5+SS
RQ value	1	0.778	0.931	2.517	1.716	1.306
CT value						
miR-375-3p	24.474	23.718	23.945	24.422	23.357	23.701
	24.456	23.716	23.967	24.435	23.333	23.688
u6	8.582	7.401	7.951	9.862	8.214	8.207
	8.509	7.468	7.915	9.820	8.196	8.115

	Four					
	Con	pEZ-MO2	pEZ-MO2-IRF5	SS	pEZ-MO2+SS	pEZ-MO2-IRF5+SS
RQ value	1	0.692	0.809	1.369	0.97	0.772
CT value						
miR-375-3p	23.225	23.003	22.845	22.694	22.641	23.500
	23.291	23.021	22.782	22.724	22.660	22.727
u6	7.493	6.643	6.708	7.394	6.869	6.972
	7.510	6.806	6.797	7.418	6.854	6.997

Fig 5 C

	OD value(570 nM)					
	HepG2			QGY-7703		
Con	0.585	0.572	0.575	0.705	0.632	0.652
pEZ-MO2	0.604	0.588	0.584	0.819	0.723	0.661
pEZ-MO2-IRF5	0.57	0.606	0.593	0.811	0.793	0.718
SS	0.339	0.344	0.36	0.439	0.436	0.433
pEZ-MO2+SS	0.396	0.402	0.384	0.473	0.455	0.452
pEZ-MO2-IRF5+SS	0.476	0.445	0.45	0.551	0.55	0.521

Fig 6 E

	Con1	con2	con3	con4	con5	con6	con7	con8	con9
CT value	1	1	1	1	1	1	1	1	1
CCAT1	14.998	15.240	16.922	16.148	15.249	15.977	15.689	15.009	15.849
	14.988	15.233	16.901	16.264	15.294	16.022	15.902	14.987	15.869
	14.888	15.326	17.017	16.239	15.272	15.938	15.818	14.950	15.816
GAPDH	15.380	14.959	17.295	16.770	15.587	16.270	15.967	14.916	15.895
	15.097	15.606	17.516	16.767	15.659	16.578	16.338	15.169	15.798
	15.590	15.620	17.636	16.917	15.767	16.528	16.367	15.434	15.873
	L1	L2	L3	L4	L5	L6	L7	L8	L9
CT value	1.503	1.329	0.940	1.080	0.966	0.710	0.878	1.219	0.862
CCAT1	15.454	15.455	15.474	16.117	16.831	16.168	15.946	14.992	16.649
	15.387	15.463	15.417	16.313	16.975	16.134	15.996	15.108	16.721
	15.542	15.389	15.403	16.114	16.975	16.088	15.908	15.093	16.740
GAPDH	16.304	15.858	15.437	16.775	17.271	16.095	16.035	15.361	16.299
	16.552	15.897	15.873	17.034	17.234	16.153	16.178	15.413	16.701
	16.484	16.168	16.321	16.871	17.321	16.097	16.336	15.851	16.500
	H1	H2	H3	H4	H5	H6	H7	H8	H9
CT value	0.702	0.682	0.665	0.688	0.541	0.581	0.654	0.682	0.803
CCAT1	15.716	15.732	15.471	15.874	15.935	16.899	15.953	17.640	15.974
	15.834	15.779	15.463	15.964	15.898	16.857	15.914	17.690	15.959
	15.791	15.619	15.452	15.895	15.936	16.856	15.920	17.735	15.961
GAPDH	15.303	15.272	15.724	16.194	15.770	16.734	15.778	17.221	15.820
	15.875	15.288	15.487	15.675	15.203	16.468	15.668	17.302	15.605
	15.824	15.297	15.014	16.048	15.334	16.495	15.767	17.459	15.554

Fig 6 E

	Con1	con2	con3	con4	con5	con6	con7	con8	con9
CT value	1	1	1	1	1	1	1	1	1
miR-375-3p	29.359	29.457	29.162	28.536	28.877	28.957	28.025	28.493	28.078
	29.263	29.615	29.157	28.631	28.855	29.026	27.999	28.365	27.946
	29.248	29.610	29.065	28.629	28.847	28.637	28.069	28.399	27.945
u6	8.091	8.550	7.449	8.158	7.171	7.713	7.717	7.409	7.262
	8.163	8.652	7.424	8.183	7.229	7.704	7.782	7.541	7.306
	8.076	8.694	7.402	8.153	7.243	7.816	7.806	7.598	7.355
	L1	L2	L3	L4	L5	L6	L7	L8	L9
CT value	0.821	1.009	0.572	2.098	1.992	0.965	1.474	0.879	1.743
miR-375-3p	28.794	28.547	29.240	27.154	29.146	28.653	28.134	28.747	27.957
	28.829	28.753	29.146	27.131	29.270	28.644	28.338	28.676	27.952
	28.901	28.695	29.147	27.185	29.179	28.709	28.310	28.776	27.983
u6	7.432	7.548	7.305	7.729	7.509	7.379	9.648	7.867	7.873
	7.368	7.644	7.368	7.664	7.434	7.601	9.631	8.065	7.893
	7.482	7.546	7.191	7.757	7.452	7.524	9.667	8.067	7.822
	H1	H2	H3	H4	H5	H6	H7	H8	H9
CT value	1.799	1.188	1.080	1.864	1.211	1.411	1.484	1.808	1.983
miR-375-3p	28.611	28.514	28.834	28.078	28.970	28.801	28.452	28.317	27.522
	28.766	28.352	28.727	28.075	28.924	28.902	28.527	28.150	27.472
	28.613	28.381	28.801	28.080	29.105	28.764	28.446	28.281	27.227
u6	7.688	7.844	7.394	8.481	7.922	7.882	9.651	8.310	7.955
	7.837	7.717	7.372	8.495	7.851	8.071	9.648	8.089	7.944
	7.669	7.788	7.489	8.650	7.826	8.061	9.624	8.203	7.237

Fig 6 F

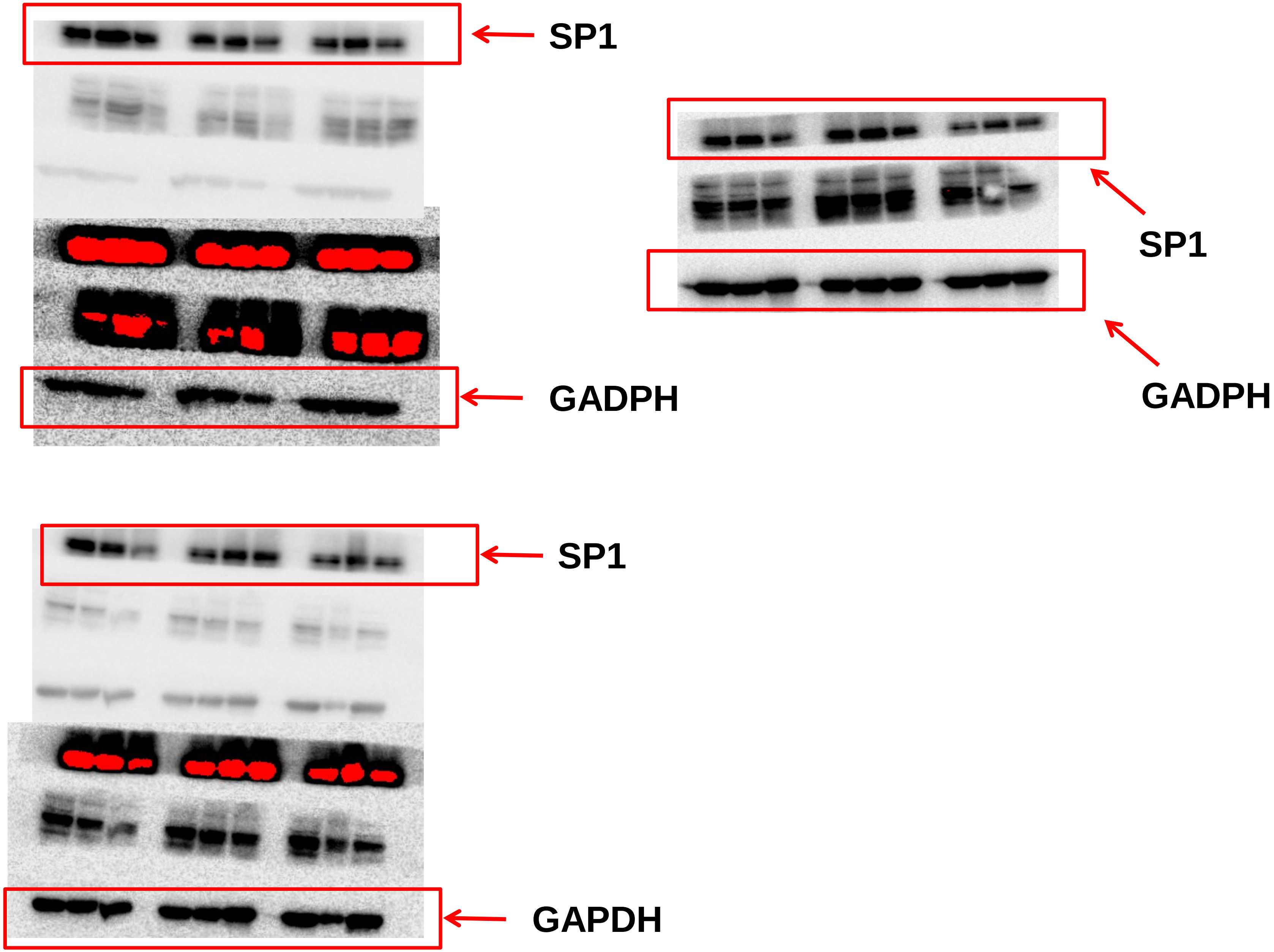

Fig 6 F

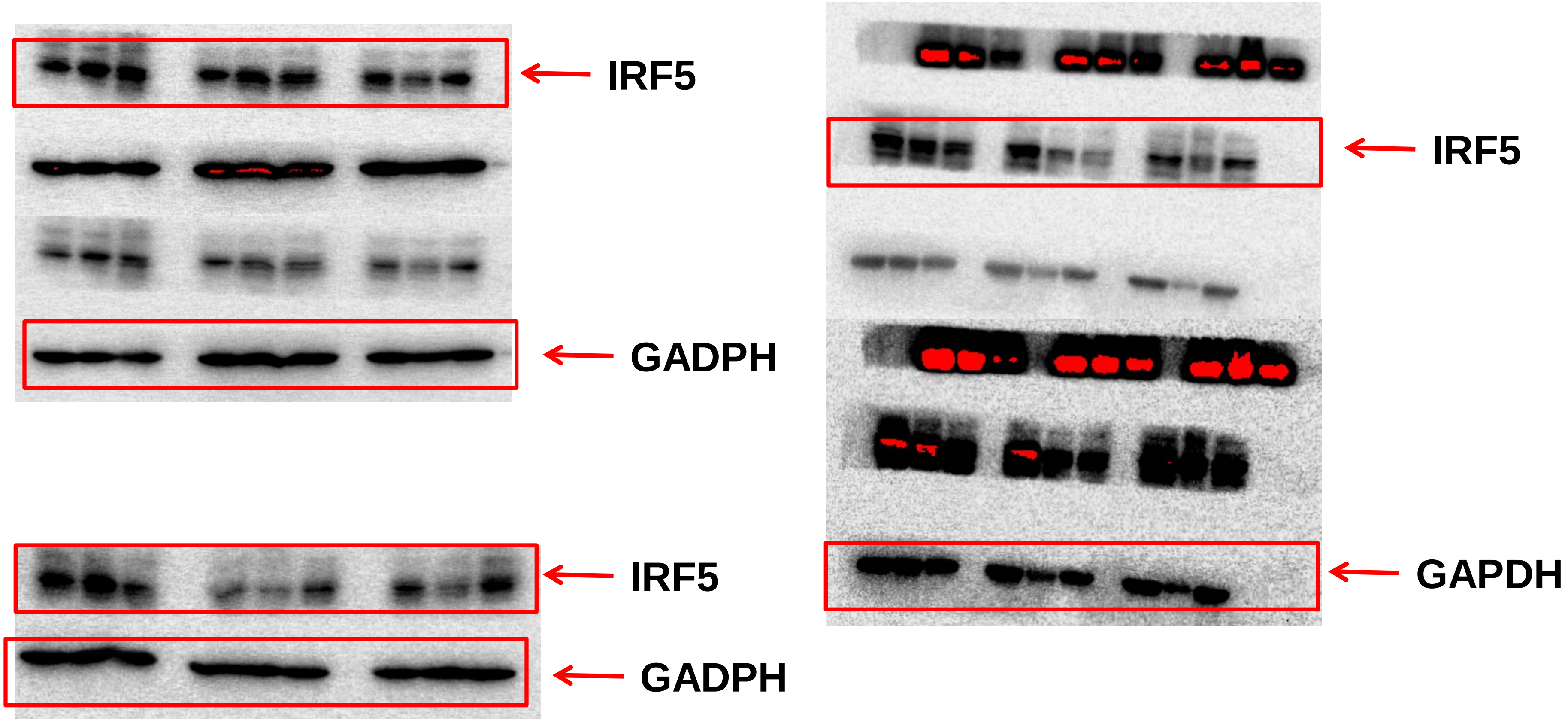

Revised data

CCAT1-miR-375-3p RIP

		CT value	CT value	CT value	CT value	—△CT				$2^{-\triangle CT}$			
	H-C-input	29.623	29.867	29.693	29.743								
	H-C-IgG	34.439	34.272	34.148	32.153	-4.817	-4.406	-4.455	-2.410	0.03548	0.04718	0.04561	0.18819
	H-C-AGO2	26.593	26.785	26.598	26.827	3.029	3.082	3.095	2.916	8.16470	8.46845	8.54369	7.54885
	H-S-input	28.785	29.567	29.201	29.355					1.00000	1.00000	1.00000	1.00000
	H-S-IgG	33.827	33.119	32.519	32.316	-5.041	-3.552	-3.318	-2.961	0.03037	0.08524	0.10026	0.12844
	H-S-AGO2	26.099	26.351	26.355	26.434	7.728	6.768	6.165	5.882	212.01991	109.01055	71.73476	58.96131
**miR-375-3p**													
	Q-C-input	19.845	19.947	19.979	19.906								
	Q-C-IgG	27.368	27.384	27.373	27.578	-7.524	-7.437	-7.394	-7.673	0.00543	0.00577	0.00595	0.00490
	Q-C-AGO2	16.827	16.881	16.899	16.936	3.018	3.066	3.079	2.970	8.10051	8.37350	8.45182	7.83485
	Q-S-input	19.499	19.638	19.544	19.618					1.00000	1.00000	1.00000	1.00000
	Q-S-IgG	25.836	25.857	25.912	25.893	-6.337	-6.219	-6.369	-6.275	0.01237	0.01342	0.01210	0.01292
	Q-S-AGO2	16.765	16.805	16.803	16.740	2.734	2.833	2.741	2.878	6.65145	7.12754	6.68586	7.35058
	H-C-input	16.976	16.815	16.840	17.032								
	H-C-IgG	23.762	23.650	24.095	24.145	-6.786	-6.834	-7.255	-7.113	0.00906	0.00876	0.00655	0.00722
	H-C-AGO2	19.823	19.766	19.831	20.172	-2.847	-2.950	-2.991	-3.140	0.13898	0.12938	0.12578	0.11345
	H-S-input	16.353	16.727	16.845	16.851								
	H-S-IgG	23.865	24.130	24.049	24.196	-7.512	-7.403	-7.205	-7.345	0.00548	0.00591	0.00678	0.00615
	H-S-AGO2	19.758	19.840	19.907	19.965	-3.405	-3.113	-3.062	-3.114	0.09438	0.11558	0.11977	0.11551
**CCAT1**													
	Q-C-input	20.956	21.169	21.315	21.070								
	Q-C-IgG	28.848	28.748	28.609	28.967	-7.891	-7.580	-7.294	-7.897	0.00421	0.00523	0.00637	0.00419
	Q-C-AGO2	20.549	20.750	20.673	20.663	0.407	0.419	0.642	0.407	1.32629	1.33670	1.56076	1.32569
	Q-S-input	21.974	22.141	22.357	22.298					1.00000	1.00000	1.00000	1.00000
	Q-S-IgG	30.119	29.881	29.800	30.289	-8.145	-7.741	-7.443	-7.991	0.00353	0.00468	0.00575	0.00393
	Q-S-AGO2	21.734	21.654	21.748	21.753	0.240	0.487	0.609	0.545	1.18134	1.40105	1.52493	1.45919

RIP -WB

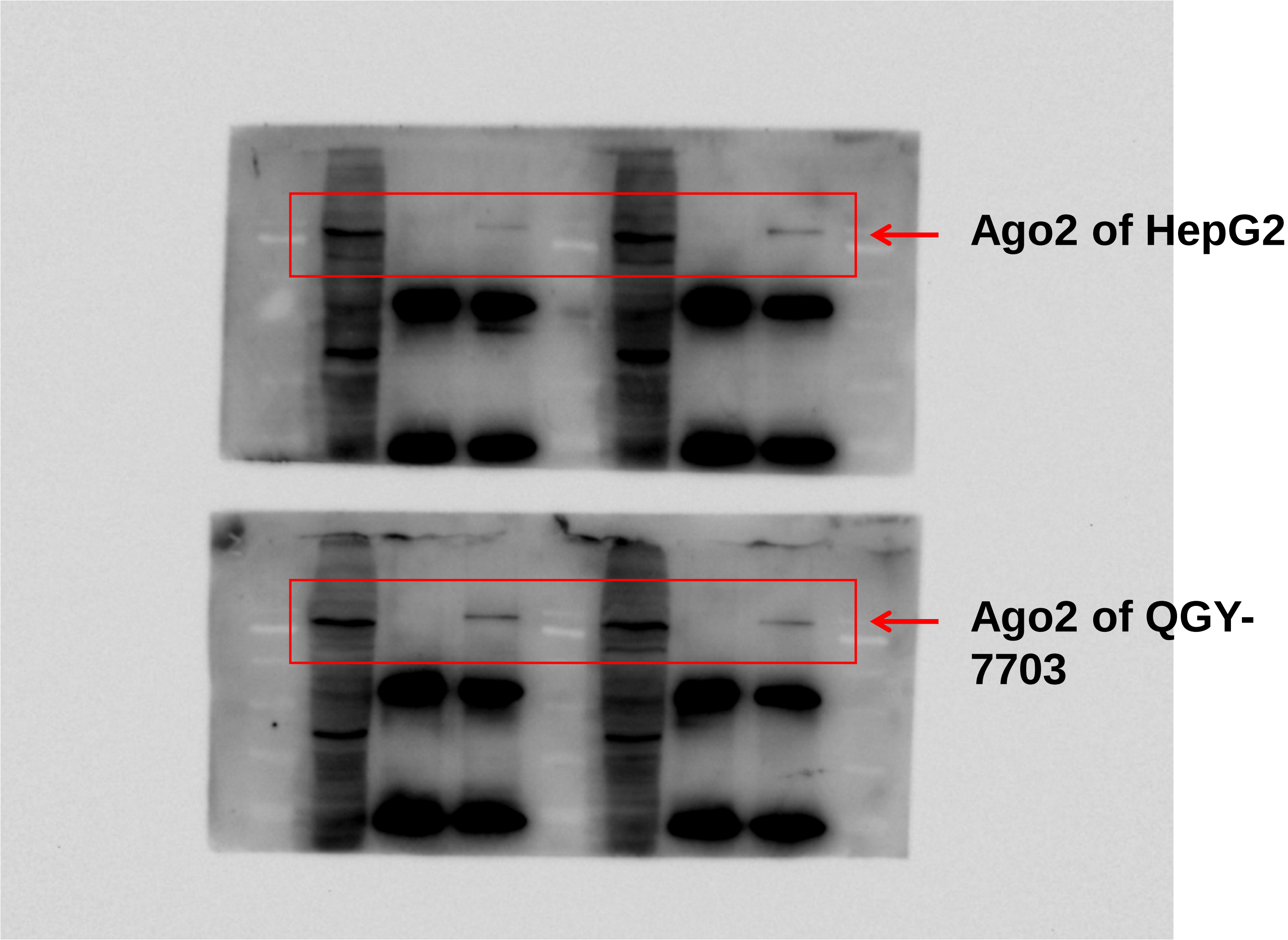

L02			OD value (570nM)												
		24H				48H					72H				
SS	0	1.028	1.037	1.02		0	1.275	1.284	1.418		0	1.77	1.743	1.78	
	5	1.043	1.037	1.069		5	1.235	1.249	1.348		5	1.637	1.65	1.654	
	10	0.888	0.902	1.019		10	1.087	1.108	1.126		10	1.369	1.382	1.437	
	20	0.907	0.887	1.038		20	1.152	1.173	1.173		20	1.451	1.441	1.41	
	30	0.982	0.869	0.886		30	1.247	1.191	1.234		30	1.246	1.242	1.377	
	40	0.665	0.674	0.682		40	1.02	0.956	0.874		40	1.143	1.152	1.115	
	50	0.653	0.673	0.632		50	0.687	0.617	0.598		50	0.723	0.706	0.725	

CCAT1 3'-UTR

Application: Tecan i-control			Tecan i-control , 1.10.4.0			
Device: infinite M1000Pro			Serial number: 1211011466			
Firmware: V_1.05_11/2011_S3LCE_ALPHA (Nov 3 2011/09.27.2			MAI, V_1.05_11/2011_S3LCE_ALPHA (Nov 3 2011/09.27.24)			
Date:	2019-9-12					
Time:	8:37:16					
System			TECAN			
User			TECAN\Administrator			
Plate			Corning 96 Flat Bottom white Polystyrol [COR96fw half area.pdfx]			
Plate-ID (Stacker)						
Shaking (Linear) Duration:			3 s			
Shaking (Linear) Amplitude:			2 mm			
Shaking (Linear) Frequency:			654 rpm			
Label: Label1						
Mode			Luminescence			
Attenuation			AUTOMATIC			
Color for OD2 Attenuation						
Integration Time			1000 ms			
Settle Time			0 ms			
Part of Plate			A7-D12			
Start Tir	2019/9/12 8:37:28					
	Temperature: 23 ° C					
<>	7	8	9	10	11	12
A	346046	515089	443928	482566	333632	335245
B	582389	437317	604034	739733	500723	633133
C	1321085	1987141	1665936	3095041	2302759	2258175
D	2123370	1962813	2057929	2609398	2417242	2459446

	HepG2			
	Gluc value	SEAP value	Gluc/SEAP	Relative to Con
CCAT1 3'-UTR WT+ Negative Con	346046	1321085	0.261941	1
	515089	1987141	0.259211	1
	443928	1665936	0.266474	1
CCAT1 3'-UTR WT+ MiR-375-3p mimics	482566	3095041	0.155916	0.595233
	333632	2302759	0.144884	0.558941
	335245	2258175	0.148458	0.557122
CCAT1 3'-UTR MUT+ Negative Con	582389	2123370	0.274276	1.047091
	437317	1962813	0.222801	0.859536
	604034	2057929	0.293515	1.10148
CCAT1 3'-UTR MUT+ MiR-375-3p mimic:	739733	2609398	0.283488	1.08226
	500723	2417242	0.207146	0.799142
	633133	2459446	0.257429	0.966059

Application: Tecan i-control | Tecan i-control , 1.10.4.0
Device: infinite M1000Pro | Serial number: 1211011466
Firmware: V_1.05_11/2011_S3LCE_ALPHA (Nov 3 2011/09.27.2 | MAI, V_1.05_11/2011_S3LCE_ALPHA (Nov 3 2011/09.27.24)

Date: 2019-9-7
Time: 9:34:51

System: TECAN
User: TECAN\Administrator
Plate: Corning 96 Flat Bottom white Polystyrol [COR96fw half area.pdfx]
Plate-ID (Stacker)

Shaking (Linear) Duration: 3 s
Shaking (Linear) Amplitude: 2 mm
Shaking (Linear) Frequency: 654 rpm

Label: Label1
Mode: Luminescence
Attenuation: AUTOMATIC
Color for OD2 Attenuation
Integration Time: 1000 ms
Settle Time: 0 ms
Part of Plate: A1-H6
Start Tim 2019/9/7 9:35:03

Temperature: 23.7 ° C

<>	1	2	3	4	5	6
A	365385	348002	356247	318591	303747	359055
B	413201	392835	395312	438490	447470	463741
C						
D	670234	947007	827266	846669	983895	1050128
E	1007901	1087600	934623	1016380	1176745	1046227

	QGY-7703			
	Gluc value	SEAP value	Gluc/SEAP	Relative to Con
CCAT1 3'-UTR WT+ Negative Con	365385	670234	0.54516	1
	348002	947007	0.367476	1
	356247	827266	0.430632	1
CCAT1 3'-UTR WT+ MiR-375-3p mimics	318591	846949	0.376163	0.690005
	303747	983895	0.308719	0.840107
	359055	1050128	0.341915	0.793986
CCAT1 3'-UTR MUT+ Negative Con	413201	1007901	0.409962	0.752002
	392835	1087600	0.361194	0.982907
	395312	934623	0.422964	0.982194
CCAT1 3'-UTR MUT+ MiR-375-3p mimic:	438490	1016380	0.431423	0.79137
	447470	1176745	0.380261	1.034792
	463741	1046227	0.443251	1.029304
