# Supplementary material for: The Reciprocal Interaction Between LncRNA CCAT1 and miR-375-3p Contribute to the Downregulation of IRF5 Gene Expression by Solasonine in HepG2 Human Hepatocellular Carcinoma Cells
Source: Front Oncol. 2019 Oct 18;9:1081. doi: 10.3389/fonc.2019.01081 (PMC6813207; doi:10.3389/fonc.2019.01081)
Supplement: Supplementary file 2 [file Data_Sheet_2.PDF]

Report of Human Cell Line Authentication

Ⅰ. Sample

Sample Name: labeled as ‘Hep G2’

Ⅱ. Method and Procedure

1. PCR is amplified with STR Multi-amplification Kit (PowerPlex™21D System);
2. PCR products are assayed with 3100 DNA Analyzer (Applied Biosystems®).
3. Amplification of gene COX1 and electrophoresis are employed to survey the species of the sample.

Ⅲ. Results

1. The STR profiles of the cell line sample are in the attached table and figure.
2. The search result in ATCC and DSMZ databases.
3. The electrophoresis figure of gene COX1.

Hep G2: ①No loci has tri-alleles or tetra-alleles. Contamination of other human cell lines are not found (Figure 1 & Table 1). ②Compared the STR data of HEPG2 cell line in the databases of ATCC and DSMZ, all the alleles of HEPG2 were exactly matched with the alleles of HEPG2 cells found in both cell banks (Figure 2&3). ③The sample is a human cell line. Contamination of other species cells are not found in the sample (Figure 4).

To all above, the sample is a single cell line, and it is HEPG2 cell line.

Operator: Xiaohua Mo　　　　Auditor: Xuanyi Liang

Guangzhou Cellcook Biotech Co., Ltd

GUANGZHOU CELLCOOK BIOTECH CO., LTD
26 AUG 2019
CELL STR PROFILING

（Notice:This authentication report is restricted to the cell sold from Guangzhou Cellcook Biotech Co., Ltd, and the date with seal is the date of delivery.）

Figure 1. STR profiles of Hep G2 cell line

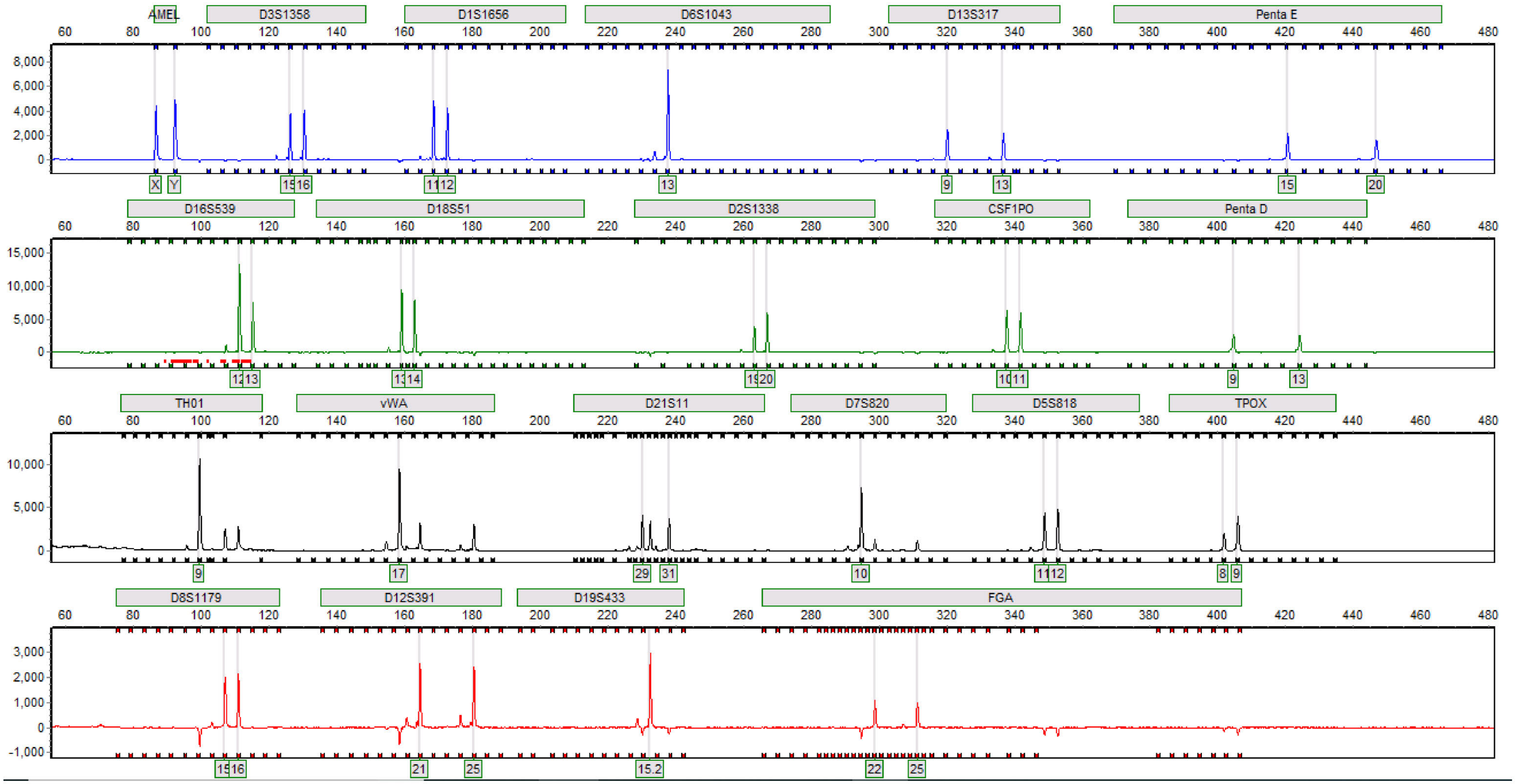

Table 1. STR profiles of Hep G2 cell line

21	Allele1	Allele2
AMEL	X	Y
D3S1358	15	16
D1S1656	11	12
D6S1043	13	
D13S317	9	13
Penta E	15	20
D16S539	12	13
D18S51	13	14
D2S1338	19	20
CSF1PO	10	11
Penta D	9	13
TH01	9	
vWA	17	
D21S11	29	31
D7S820	10	
D5S818	11	12
TPOX	8	9
D8S1179	15	16
D12S391	21	25
D19S433	15.2	
FGA	22	25

Figure 2. Search result in ATCC database

SEARCH THE STR DATABASE

As part of our continuing efforts to characterize and authenticate the cell lines in the Cell Biology collection, ATCC has developed a comprehensive database of short tandem repeat (STR) DNA profiles for all of our human cell lines. View our brief tutorial before starting.

1. STR Profiling Analysis
2. Matching Algorithm
3. Interrogating the Database

Showing 1 - 4 Of 4

PageSize: 100

Add to Cart	%Match	ATCC® Number	Designation	D5S818	D13S317	D7S820	D16S539	vWA	TH01	AMEL	TPOX	CSF1PO
☐	100.0	HB-8065	Hep G2Hepatocellular CarcinomaHuman	11,12	9,13	10	12,13	17	9	X,Y	8,9	10,11
☐	100.0	CRL-11997	HEP G2/2.2.1 CELL LINE	11,12	9,13	10	12,13	17	9	X,Y	8,9	10,11
☐	100.0	MRA-975	HC-04	11,12	9,13	10	12	17	9	X,Y	8,9	10,11
☐	93.0	CRL-10741	C3A (HepG2/C3A) Hepatocellular CarcinomaHuman	11,13	9,13	10	12,13	17	9	X,Y	8,9	10,11

Add to Cart | Export to Excel

Figure 3. Search result in DSMZ database

Result of STR matching analysis by your data.

- DSMZ Profile Database -

A graphical presentation is shown at the bottom of this page.

EV	Cell No.	Cell name	Locus names									Figures
			D5S818	D13S317	D7S820	D16S539	VWA	TH01	AM	TPOX	CSF1PO	
	Query (Your Cell)		*11,12*	*9,13*	*10,10*	*12,13*	*17,17*	*9,9*	*X,Y*	*8,9*	*10,11*	
1.00(36/36)	180	HEP-G2	11,12	9,13	10,10	12,13	17,17	9,9	X,Y	8,9	10,11	-
1.00(36/36)	CRL-11997	HEP G2/2.2.1	11,12	9,13	10,10	12,13	17,17	9,9	X,Y	8,9	10,11	-
1.00(36/36)	HB-8065	Hep G2	11,12	9,13	10,10	12,13	17,17	9,9	X,Y	8,9	10,11	-
1.00(36/36)	HB-8065.1	HepG2/SF	11,12	9,13	10,10	12,13	17,17	9,9	X,Y	8,9	10,11	-
1.00(36/36)	JCRB1054	Hep G2	11,12	9,13	10,10	12,13	17,17	9,9	X,Y	8,9	10,11	-
1.00(36/36)	RCB1648	Hep G2	11,12	9,13	10,10	12,13	17,17	9,9	X,Y	8,9	10,11	-
1.00(36/36)	RCB1886	Hep G2	11,12	9,13	10,10	12,13	17,17	9,9	X,Y	8,9	10,11	-
0.94(34/36)	CRL-10741	C3A [HepG2/C3A, derivative of Hep G2 (ATCC HB-8065)]	11,13	9,13	10,10	12,13	17,17	9,9	X,Y	8,9	10,11	-
0.94(34/36)	RCB1681	GS-HepG2	11,12	9,13	10,10	12,12	17,17	9,9	X,Y	8,9	10,11	-
0.67(24/36)	12	JVM-2	11,12	11,13	10,11	12,13	17,17	6,9	X,X	8,11	11,11	-
0.67(24/36)	417	HN	12,12	10,10	10,10	12,13	17,17	9,9	X,X	9,11	11,11	-
0.67(24/36)	669	UPCI-SCC-154	11,12	9,12	9,10	13,13	17,17	7,7	X,Y	8,9	10,12	-
0.67(24/36)	748	U-CH1	11,12	11,13	9,12	12,13	17,17	7,7	X,Y	8,11	10,11	-

Figure 4. Authentication of the species of the sample

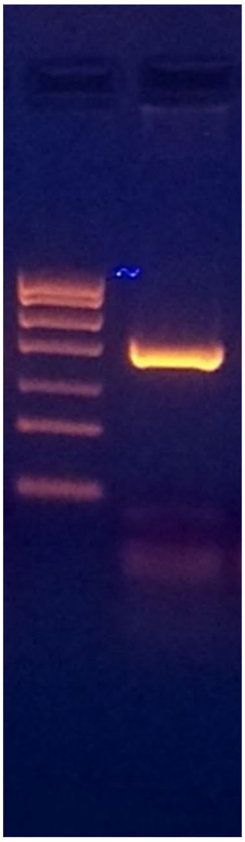

M: Marker. As the size of 700, 600, 500, 400, 300, 200 and 100bp from up to down.

Nine species are checked, as follow: *Homo sapiens* 391bp, *Cricetulus griseus*315bp, *Macaca mulatta*287bp, *Cercopithecus aethiops*222bp, *Rattus norvegicus*196bp, *Canis familiaris*172bp, *Mus musculus*150bp, *Bos Taurus*102bp, IC 70bp

The sample: The band size is 391bp which matches the size of human.
